# A Self-Assembling Senolytic Prodrug with Enhanced Bioavailability and Selective Activation for Targeting Senescent Retinal Pigment Epithelium

**DOI:** 10.34133/bmr.0261

**Published:** 2025-10-01

**Authors:** Haewon Ok, Hyun-Seo Park, Jungin Park, Sunyoung Hwang, Jiwon Jang, Jiye Kim, Gaeun Park, Dojoon Park, Tae-Eun Park, Chaekyu Kim, Ja-Hyoung Ryu

**Affiliations:** ^1^ Department of Chemistry, Ulsan National Institute of Science and Technology (UNIST), Ulsan 44919, Republic of Korea.; ^2^ Fusion Biotechnology Inc., Ulsan 44919, Republic of Korea.; ^3^ Department of Biomedical Engineering, Ulsan National Institute of Science and Technology (UNIST), Ulsan 44919, Republic of Korea.; ^4^ Department of Orthopedic Surgery, St. Vincent’s Hospital, College of Medicine, The Catholic University of Korea, Suwon-si Gyeonggi-do 442-723, Republic of Korea.

## Abstract

Senolytic therapy, which targets and selectively eliminates senescent cells, has emerged as a promising strategy for treating various age-related diseases. However, its clinical application is often limited by poor bioavailability, off-target toxicity, and the need for invasive administration routes. To overcome these challenges, we developed N201-gal, a novel β-galactosidase-reactive senolytic prodrug that self-assembles into stable nanoparticles, enabling oral administration and improved systemic bioavailability. Once internalized by senescent cells, N201-gal responds to β-galactosidase overexpression, triggering controlled drug release and inducing selective apoptosis in senescent cells while sparing normal cells. The nanoparticle formulation exhibited favorable physicochemical properties, including uniform particle size and pH stability suitable for gastrointestinal absorption. In vitro study shows that N201-gal demonstrated potent senolytic activity and reduced the expression of senescence-associated markers in retinal pigment epithelial (RPE) cells. In addition, in vivo study also shows that oral administration of N201-gal in a mouse model of doxorubicin-induced retinal senescence model significantly restored retinal tissue integrity and visual function through the targeted clearance of senescent cells. These findings highlight the potential of self-assembling senolytic prodrugs as a noninvasive and targeted therapeutic platform for age-related degenerative diseases.

## Introduction

Age-related macular degeneration (AMD), a leading cause of vision loss in older adults [1], is a multifactorial retinal disease, and cellular senescence is one of several contributing factors involved in its progression [2]. These senescent cells (SnCs) secrete inflammatory cytokines, growth factors, and proteases, known as the senescence-associated secretory phenotype (SASP) [3]. The SASP induces chronic inflammation [4] and tissue degeneration, contributing to the visual impairment characteristic of AMD [5]. Current AMD treatments focus primarily on symptom management and disease progression inhibition, with intravitreal injections and laser therapy offering only temporary relief without addressing the underlying cause of SnC accumulation [6]. This limitation necessitates repeated and invasive treatment and has spurred significant research into senolytic agents, which selectively induce apoptosis in SnCs [7], potentially alleviating chronic inflammation and tissue degeneration [8]. Nevertheless, the clinical implementation of senolytic agents faces several challenges, including insufficient selectivity for SnCs, poor bioavailability [9] of hydrophobic compounds, and a lack of effective oral administration methods. Overcoming these obstacles requires the development of novel senolytic drugs with enhanced SnC selectivity, improved water solubility, and increased oral bioavailability.

A prodrug strategy targeting senescence-associated β-galactosidase (SA-β-gal), which is overexpressed in SnCs [10], represents an attractive approach for developing oral senolytic drugs. This strategy allows for the design of compounds that are selectively activated in cells with high SA-β-gal activity, minimizing unintended effects on normal cells. The conjugation of galactose to senolytic compounds offers several advantages: It can overcome the insolubility issues often associated with senolytic drugs [11]. The numerous hydroxyl groups in galactose increase the hydrophilicity of the drug, improving its solubility [12]. Enhanced hydrophilicity leads to improved gastrointestinal absorption and significantly increased bioavailability when the drug is administered orally [13]. This approach provides a noninvasive delivery route [14], potentially increasing patient compliance and facilitating long-term treatment. Consequently, galactose-based prodrug approaches can lead to the development of orally [15] available senolytic drugs that specifically target SnCs. These novel therapeutics have the potential to revolutionize the treatment of age-related diseases by providing a more targeted, efficient, and patient-friendly approach to eliminating SnCs.

**Fig. 1.**
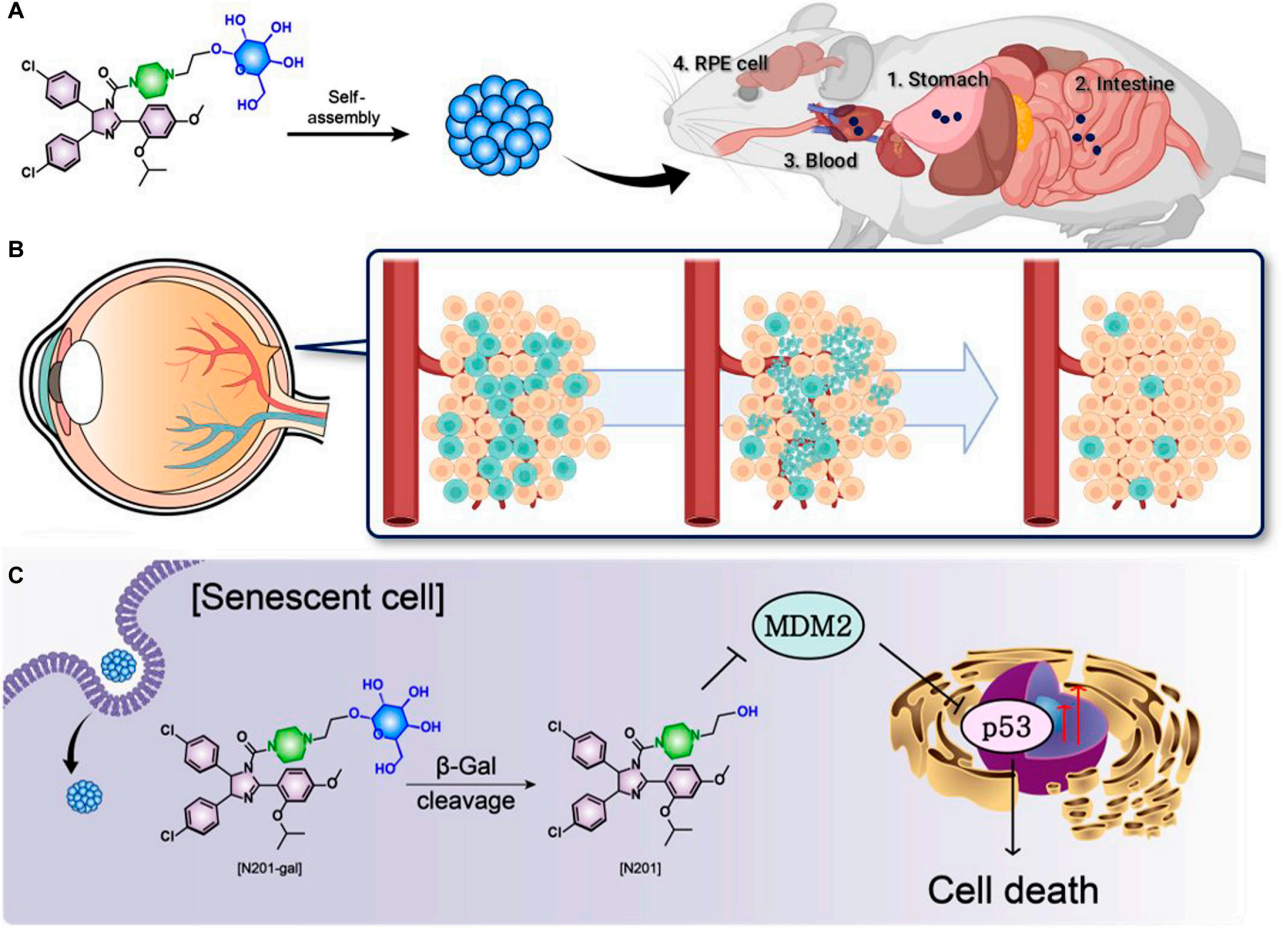
(A) Chemical structure of the oral senolytic drug series Nutlin and delivery pathway of N201-gal to retinal pigment epithelial (RPE) cells. (B) Senolytic therapy in the RPE cell line to eliminate senescent cells (SnCs). (C) Mechanism by which N201-gal induces cell death in the senescent RPE cell line.

Our approach leverages the potential of Nutlin-3a, a potent MDM2 inhibitor known to induce apoptosis in SnCs by up-regulating p53 protein expression [16]. Nutlin-3a increases p53 levels by disrupting the p53–MDM2 interaction, thereby promoting apoptosis in SnCs [17]. However, the clinical application of Nutlin-3a has been constrained by inadequate bioavailability and a lack of selectivity when it is administered orally. To overcome these limitations, we developed a self-assembling nanostructure formulation of N201-gal designed to enhance gastrointestinal absorption and selectively activate senescent RPE cells [12]. Specifically, we developed a modified version of Nutlin-3a, designated N201, which retains the central piperazine structure of Nutlin-3a while incorporating hydroxyl groups (-OH) at the terminal positions. Furthermore, we conjugated N201 with galactose, resulting in N201-gal, which improved oral bioavailability and enhanced target specificity for SnCs. N201-gal is formulated as a nanoparticle to facilitate efficient absorption through the gastrointestinal tract following oral administration. Upon reaching systemic circulation, these nanoparticles target SnCs in the retina, where N201 is activated by SA-β-gal, which is overexpressed in SnCs. The activated N201 then binds to MDM2, resulting in increased p53 up-regulation. When p53 expression exceeds a certain threshold, apoptosis is induced in SnCs (Fig. 1) [18]. Rather than replicating the full pathological complexity of AMD, this study employs a doxorubicin hydrochloride (DOX)-induced senescence model in RPE cells to examine the selective pro-apoptotic efficacy of a β-galactosidase-responsive senolytic prodrug. In addition, the ultimate goal of this research is to address senescence-associated pathology, which may be relevant to AMD and similar conditions. The utilization of N201-gal represents a potentially transformative approach for senolytic therapy, with possible implications for AMD treatment, offering a more practical and effective therapeutic option. The development of such senolytic therapies is expected to open new avenues for the treatment of age-related diseases.

## Materials and Methods

### Materials and characterization

All reagents for organic synthesis were obtained commercially without purification. DOX was from Tocris Bioscience (#2252, Bristol, UK). 2,3,4,6-Tetra-o-acetyl-α-d-galactopyranosyl bromide was purchased from Combi-Blocks. Nutlin intermediate was from Daegu-Gyeongbuk Medical Innovation Foundation. 2-Hydroxyalcohol, d-galactose, 1-(2-hydroxy ethyl) piperidine, silver carbonate, triethylamine, triphosgene, pepsin, and pancreatin were from Sigma-Aldrich. The solvents were purchased from SAMCHUN Chemicals. Senescence β-Galactosidase Staining Kit was from Cell Signaling. All fluorescent dyes [secondary fluorescence 647, LysoTracker, calcein-AM, annexin V-FITC (fluorescein isothiocyanate)] were purchased from Thermo Fisher. Western blot image was obtained using Universal Hood III by Bio-Rad. MTT [3-(4,5-dimethylthiazol-2-yl)-2,5-diphenyltetrazolium bromide] assay was performed using SpectraMax M5e Multi Mode Microplate Reader by Molecular Devices. Matrix-assisted laser desorption/ionization–time-of-flight (MALDI-TOF)/TOF (Ultraflex III, Bruker) was exploited to confirm the mass/charge ratio (*m*/*z*) of synthesis drugs. Bio-TEM (transmission electron microscopy) (JEM 1400) was exploited to obtain TEM images. Confocal laser-scanning microscopy (LSM 980) was used to obtain CLSM (confocal laser scanning microscope) images.

### Methods

#### Critical micelle concentration

The critical micelle concentration (CMC) was measured via the steady-state fluorescence pyrene emission method. Different concentrations of N201-gal solution were prepared in DI (deionized) water. One microliter of pyrene solution in ethanol (2 mM) was added to 999 μl of N201-gal solution. A solution containing 2 μM pyrene was analyzed via fluorescence spectroscopy. The excitation wavelength was set to 331.0 nm. The slit width for excitation and emission was set to 2.5 nm, and the scan speed was set to 240 nm/min. The ratio of the *I*_1_ band to the *I*_3_ band for pyrene was plotted to obtain the critical aggregation concentration (CAC).

#### Fluorescence polarization binding assays

Fluorescence polarization (FP) assays were conducted via a Synergy 2 Multi-Mode Microplate Reader (BioTek) with excitation and emission filters set to 485 and 528 nm, respectively. The fluorescence intensities parallel (intparallel) and perpendicular (intperpendicular) to the plane of excitation were recorded in U-bottom black 96-well microplates (Greiner, 650209) at room temperature (RT) (approximately 20 °C). Background fluorescence from blank samples containing the reference buffer was subtracted. Steady-state FP was calculated via the equation *p* = (Intparallel − GIntperpedicular)/(Intparallel + GIntperpedicular), with a correction factor 𝐺 (empirically determined to be 0.998) to account for differences in the transmission of vertically and horizontally polarized light. All FP values are reported in millipolarization units. FP assays were performed in a buffer consisting of 10 mM Hepes (pH 7.4), 150 mM NaCl, 0.05% (v/v) Tween 20, and 0.5 mM dithiothreitol. First, 12.5 μM of the FITC-p53 peptide (Anaspec Inc., AS-62386) and 100 nM of MDM2 (Abcam, ab167941) were mixed and then reacted for 1 h. They were mixed with a series of diluted N201 and N201-gal compounds (used at concentrations ranging from 0 to 10 μM) in triplicate in a final volume of 50 μl, and FP was measured after the mixture was incubated for 1 to 3 h. The FP values were plotted against the log of the inhibitor concentration via a nonlinear regression model. The IC_50_ (median inhibitory concentration) was calculated from the dose–response–inhibition model in GraphPad Prism.

#### Making simulated gastric fluid and intestinal fluid

The artificial gastric juice was prepared according to a method described in the United States Pharmacopeia. The solution was prepared by dissolving 2.0 g of sodium chloride and 3.2 g of pepsin in 500 ml of distilled water. Subsequently, 7.0 ml of hydrochloric acid (30%) was added, and the volume was adjusted to 1 l with distilled water. Finally, the pH was adjusted to 1.2 with 1 N hydrochloric acid. Artificial small intestinal fluid was prepared by dissolving 6.8 g of KH₂PO₄ in 250 ml of distilled water, followed by the addition of 190 ml of 0.2 N NaOH and 400 ml of distilled water. Subsequently, 10 g of pancreatin was added, resulting in a total volume of 1 l. The final pH was adjusted to 7.5 with 0.2 N NaOH.

#### Enzymatic cleavage assay

Enzymatic cleavage of N201-gal was carried out with β-galactosidase (12.1 units/mg protein) from Sigma-Aldrich. N201-gal (μM) was incubated with β-galactosidase in phosphate-buffered saline (PBS) (10 mM, 1 ml, pH 7.3) at 37 °C overnight. The hydrolysis process was detected via high-performance liquid chromatography (HPLC) by monitoring the changes in the absorption of the Nutlin series at 254 nm.

#### Cell culture

A human retinal pigment epithelial cell line (ARPE-19) was obtained from American Type Culture Collection (ATCC), and Caco-2 cells were obtained from the Korean Cell Line Bank. ARPE-19 cells were cultured in Dulbecco’s modified Eagle’s medium:nutrient mixture F12 (DMEM/F12) containing 10% fetal bovine serum (FBS) (Life Technologies) and 1% penicillin/streptomycin (Life Technologies) at 37 °C in a humidified atmosphere with 5% CO_2_. Caco-2 cells were cultured in DMEM (LM001-51; Welgene) supplemented with 10% FBS (Life Technologies) and 1% penicillin/streptomycin (Life Technologies) at 37 °C in a humidified atmosphere with 5% CO_2_. All the following cellular experiments were performed in triplicate unless otherwise noted.

#### The cellular senescence model was established as follows

ARPE-19 cells were seeded into 6-well plates at a density of 200,000 cells per well prior to the induction of cellular senescence with DOX (Tocris Bioscience, Bristol, UK, #2252). After 24 h, the cells were treated with 250 nM DOX for 3 d. After 3 d, the cells were placed in DOX-free medium, which was changed daily. At 10 d, DOX-treated SnCs and non-SnCs (only the culture medium was changed) were seeded in 24- or 6-well plates for further experiments.

#### SA-β-gal assay

Senescence was assessed via an SA-β-gal staining kit according to the manufacturer’s protocol (#9860, Cell Signaling Technology, Danvers, MA, USA). Briefly, RPE/choroid tissues were fixed for 30 min in 1× fixative solution, washed with 1× PBS, and incubated overnight with SA-β-gal staining solution at 37 °C. To measure SA-β-gal, the dense pigment in the RPE was bleached with 10% H_2_O_2_, incubated for 45 min on a 55 °C heat block, and rinsed with PBS. The RPE/choroid tissues were then flat-mounted via an optical microscope (Olympus SZ51) (#18,606–20; Polysciences Inc., Warrington, PA, USA). Images of the stained RPE/choroid flat mounts were captured via an inverted microscope (Carl Zeiss Axio Scope A1, Gottingen, Germany). For SA-β-gal staining of ARPE-19 cells, ARPE-19 cells were rinsed with 1× PBS once. Then, the ARPE-19 cells were allowed to fix for 10 to 15 min at RT with 1× fixative solution and washed with 1× PBS 2 times. Then, β-galactosidase staining solution was added to the ARPE-19 cells, and the cells were incubated overnight. Inverted fluorescence microscopy (IX71, Olympus) was used to observe the stained SA-β-gal.

#### MTT assay

For the MTT assay, the cells were seeded in a 96-well plate (Thermo Fisher Scientific Inc.) at a density of 10,000 per well. After growing for 24 h, different concentrations of Nutlin-3a, N201, and N201-gal solutions (0, 1, 5, 10, 20, 30, 50, and 100 μM) were incubated with the cells. After 24 h, 100 μl of MTT solution diluted 10 times with medium was replaced for 4 h, and MTT was solubilized with 100 μl of sodium dodecyl sulfate (SDS)–HCl solution for 12 h. The MTT absorbance at 595 nm was measured with a microplate reader. All the experiments were performed in triplicate.

#### Organelle colocalization assay

ARPE-19 and SnC ARPE-19 cells were seeded in an 8-well Lab-Tek II slide chamber at a density of 1 × 10^4^ per well. After 24 h of growth, 20 μM FITC-N201-gal solution diluted in cell culture medium was added, and the mixture was incubated for 2 h. Then, the medium was removed, and the cells were incubated with LysoTracker Blue DND-22 (Invitrogen) following the manufacturer’s protocol. The cells were washed with PBS and analyzed via an LSM 980 instrument (Zeiss). The histogram for fluorescence intensity was obtained from ImageJ software.

#### Immunofluorescence assay

ARPE-19 and SnC ARPE-19 cells were seeded in an 8-well Lab-Tek II slide chamber at a density of 1 × 10^4^ per well. After 24 h of growth, the cells were washed with PBS and fixed in 4% paraformaldehyde for 30 min at 37 °C. The cells were washed with PBS 3 times for 5 min each. Immunostaining was performed after permeabilization in PBS with 0.1% Triton X-100 at RT and blocking for 1 h at 4 °C in 1% bovine serum albumin (BSA) in PBS. The cells were incubated overnight at 4 °C with p21 antibody and p53 antibody as primary antibodies. After overnight incubation, the cells were washed with PBS 3 times and incubated for 1 h with secondary fluorescence 647 at 4 °C. After further washing with PBS, the cell nuclei were stained with 4′,6-diamidino-2-phenylindole (DAPI). The stained cells without primary antibody were considered negative. Images were analyzed via an LSM 980 instrument by Zeiss.

#### Western blot

For Western blotting, whole-cell protein extracts were obtained via 1× cell lysis buffer (Cell Signaling Technology, #9803). The total protein concentration was determined by using a bicinchoninic acid (BCA) protein assay kit (Pierce) according to the manufacturer’s instructions. A total of 20 μg of protein was heated for 10 min at 95 °C, resolved via a 4% to 15% TGX SDS–polyacrylamide gel electrophoresis (PAGE) gel system (Bio-Rad), and transferred to a 0.45-μm polyvinylidene difluoride (PVDF) membrane by electroblotting. The PVDF membranes were blocked with TBST (tris-buffered saline with Tween 20) 5% milk at RT for 1 h and probed overnight at 4 °C with primary antibodies. We used the following primary antibodies: anti-p53 (sc-126, 1:1,000), anti-p21 (sc-817, 1:2,000), anti-MDM2 (ab259265, 1:1,000), anti-HMGB1 (ab18256, 1:10,000), and anti-GAPDH (glyceraldehyde-3-phosphate dehydrogenase) (sc-32233, 1:10,000). Protein levels were visualized after incubation with the appropriate secondary antibodies conjugated with horseradish peroxidase, followed by detection with Super Signal West Pico Plus ECL (#34580, Pierce). The signals were detected via an automated imaging system (ChemiDoc; Bio-Rad Laboratories, Hercules, CA).

#### For the cell apoptosis assay

The DOX-induced senescent ARPE-19 cells were seeded at a density of 2 × 10^5^ cells/well in 6-well dishes and incubated overnight at 37 °C under 5% CO_2_. The cells were treated with 100 μM Nutlin-3a, N201, or N201-gal for 24 h. After that, the cells were washed, trypsinized, and collected by centrifugation. The cells were resuspended by washing with 1× annexin-binding buffer and collected by centrifugation. Annexin-V and propidium iodide (PI) staining were performed by adding 5 μl of annexin V (stock concentration of 200 μg/ml) and 1 μl of PI (stock concentration of 100 μg/ml) to each 100 μl of cell suspension with 1× annexin-binding buffer and incubating for 15 min at RT in the dark. After that, 400 μl of annexin-binding buffer was added to the cell suspensions. The cells were analyzed by a BD FACSVerse flow cytometer using emission filters at 530 and 610 nm.

#### For the live-dead cell assay

The SnC ARPE-19 cell line was seeded in an 8-well Lab-Tek II slide chamber at a density of 1 × 10^4^ per well. Nutlin-3a, N201, and N201-gal were subsequently added to the cells in DMEM-F12 medium supplemented with 10% FBS at 37 °C. The cells were incubated for 24 h and washed 3 times with PBS to remove the remaining drugs. Then, the samples were incubated with calcein-AM (2 μM) at 37 °C for 1 h, and PI (4.5 μM) was added, after which they were imaged via a confocal microscope.

#### Caco-2 transwell model

Caco-2 cells were purchased from the Korean Cell Line Bank. The cells were cultured in DMEM (LM001-51; Welgene) supplemented with 10% FBS (TMS-013-BKR; Merck Millipore) and 1% penicillin–streptomycin solution (LS202-02; Welgene) at 37 °C in a 5% CO_2_ humidified incubator. Before Caco-2 cells were seeded onto the Transwell insert (6.5 mm inner diameter, 0.33 cm^2^, 0.4 μm pore size, 3470; Corning, NY, USA), the polyester membranes were coated with 200 μg/ml rat tail collagen type I (354236; Corning) and 1% Matrigel (354230; Corning) in DMEM and then incubated in a humidified 37 °C incubator for 1 h. After the membranes were washed with complete medium, Caco-2 cells (passage numbers 37 to 44) were seeded at 5 × 10^5^ cells/cm^2^ on the apical side with 0.2 ml of complete medium, whereas 0.7 ml of the same completed medium was added to the basal compartment of the Transwell insert. The medium was replaced every 24 h for 6 d. After 6 d in culture, the Caco-2 monolayers were subjected to a permeability assay after the transepithelial electrical resistance (TEER) value was measured. The integrity of the tight junction barrier in the Caco-2 model was evaluated by measuring the TEER across the epithelial cell monolayer via an EVOM2 (World Precision Instruments, Sarasota, FL, USA) instrument equipped with an STX2 electrode (World Precision Instruments, Sarasota, FL, USA). A TEER value greater than 300 Ω∙cm^2^ was considered appropriate for using the cell monolayer in transport experiments. At the end of the transport experiments, the TEER was assessed to evaluate the integrity of the Caco-2 monolayer.

#### In vitro intestinal epithelial permeability assay

Caco-2 transwell models were rinsed 3 times with Hanks’ balanced salt solution (HBSS) and then equilibrated in HBSS at 37 °C for 1 h. N201 and N201-gal were dissolved in dimethyl sulfoxide (DMSO) and diluted in HBSS to obtain a final DMSO concentration of 0.1%. After equilibration, 40 μl of medium was retrieved from the apical compartment, and the same volume of N201 or N201-gal solution was added at a final concentration of 10 μM to the apical compartment. For the permeability assay, the Caco-2 transwell models were placed on an orbital shaker at 50 rpm at 37 °C. Samples (100 μl) were collected from the basal compartment at 0.5, 1, and 2 h and replaced with an equal volume of the same buffer. To inhibit adenosine triphosphate (ATP)-driven endocytosis, the cells were preincubated at 4 °C for 1 h, and a permeability assay was also conducted at 4 °C. The fluorescence intensity of N201 and N201-gal conjugated with FITC was measured by a microplate reader, and the apparent permeability coefficient (*P*_app_) was calculated according to the following equation:

$$P_{\text{app}}=\text{DQ}1/\left(A\times C_{0}\times \text{DT}1\right)$$ (1)

where DQ1 is the amount in the receiver compartment, A is the surface area, $C_{0}$ is the initial concentration in the donor compartment, and DT1 is the incubation time.

#### Energy dependence of cellular uptake

Caco-2 transwell systems were rinsed 3 times with HBSS and then equilibrated with HBSS at 37 °C for 1 h. To inhibit ATP-driven endocytosis, the cells were preincubated at 4 or 37 °C as a control for 1 h. Samples from the apical compartment (40 μl) were subsequently collected, and the same volume of N201-gal solution was added at a final concentration of 10 μM to the apical chamber. During the assay, the Caco-2 transwell systems were incubated on an orbital shaker at 50 rpm at 4 or 37 °C for an additional 30 min. Finally, the samples were analyzed via a microplate reader (SYNERGY neo2; BioTex).

#### Immunofluorescence assay of Caco-2 cells

The cells were fixed with 4% paraformaldehyde for 10 min at RT. The cells were subsequently incubated in permeabilization and blocking buffer with 0.1% Triton X-100 (X100; Sigma-Aldrich) and 10% goat serum (16210064; Thermo Fisher Scientific Inc.) dissolved in Dulbecco’s PBS for 1 h. The samples were incubated with an anti-villin antibody (sc-58897, 1:100; Santa Cruz Biotechnology) overnight at 4 °C. The samples were subsequently incubated with goat anti-mouse immunoglobulin G (IgG)–Alexa Fluor 647 (A32728, 1:200; Invitrogen) for 2 h at RT in the dark. Nuclei were stained with DAPI (D9542, 1 μg/ml; Sigma-Aldrich). Images were captured via an LSM 980 immunofluorescence microscope (Carl Zeiss Inc., Thornwood, NY, USA).

#### Animals and RPE senescence model

All the animals were cared for, and all the experiments were performed in accordance with the Association for Research in Vision and Ophthalmology (ARVO) Statement for the Use of Animals in Ophthalmic and Vision Research. All procedures were approved by the Institutional Animal Care and Use Committee (IACUC) (approval no. KU23146) at the University of Konkuk. All animals were housed in a controlled barrier facility and exposed to a 12-h light/dark cycle with free access to food and water. Before the experiment, the mice were acclimatized to the facility for 1 week and then randomly assigned to the control group or treatment group. To induce retinal senescence, a single subretinal injection of doxorubicin (100 ng/μl) was administered, following previously established methods. Briefly, 8-week-old C57BL/6J mice weighing 20 to 25 g were anesthetized with a mixture of alfaxalone (60 mg/kg) and xylazine (20 mg/kg) via intraperitoneal injection. Pupils were dilated maximally via a combination of tropicamide (1%) and phenylephrine hydrochloride (0.5%). Under a surgical microscope (Olympus SZ51, Japan), a small hole was created in the limbus via a 30-gauge sterile needle. Then, a blunt 35-gauge Hamilton syringe (Hamilton Company, USA) was inserted through the hole, and 1 μl of doxorubicin dissolved and diluted in PBS (Corning, USA) was injected into the subretinal space. Three days following doxorubicin injection, N201-gal or vehicle was intravitreally injected. Coverslips were used to enhance fundus visibility during both subretinal and intravitreal injections.

#### Fundus imaging

A TRC-50 IX camera (Topcon, Japan) linked to a digital imaging system (Nikon, Japan) was used to photograph the fundus of each mouse, and a 20D (diopter) condensing lens was mounted between the camera and the eye. Conscious mice were used to avoid anesthesia complications, such as clouding of the ocular medium and vasoconstriction. After pupil dilation with tropicamide and phenylephrine hydrochloride, color fundus photography (CFP) and autofluorescence imaging (FAF) were used to assess retinal vascular status, retinal tissue, and structural damage.

#### RPE/choroid flat mounts

The enucleated eye was dissected, the anterior segment was removed, and the retina was detached and separated from the optic nerve head under a biopsy microscope. After staining, the eyecup, consisting of the RPE, was partially quartered, with 4 radial cuts from the periphery toward the optic disc. Images were acquired via an upright microscope (Leica Microsystems, Germany) or an LSM 900 confocal microscope (Carl Zeiss, Germany).

#### SA-β-gal staining

The eyecups were stained with a Senescence β-Galactosidase Staining Kit (Cell Signaling, USA) according to the manufacturer’s instructions. Briefly, the eyecups were fixed for 1 h at RT and then incubated with the provided staining reagent at 37 °C overnight. As the RPE is rich in melanin, we used 30% H_2_O_2_ to bleach the tissue. The eyecups were incubated for 45 min on a 55 °C heat block, washed with PBS, and sealed with mounting medium. The development of a blue-green color indicated SA-β-gal-positive cells, which were observed via light microscopy. The positively stained area was quantified with ImageJ (National Institutes of Health, USA) software.

#### Mitochondrial ROS measurement

To target mitochondrial reactive oxygen species (ROS) in living cells, we utilized the fluorescent dye Mitochondrial Superoxide Indicators (Invitrogen, USA) following the manufacturer’s protocols. The labeling reaction was performed with 5 μM MitoSOX dissolved in HBSS at 37 °C for 10 min. After washing with PBS, the nuclei were counterstained with Hoechst 33342 (Invitrogen, USA) at RT for 10 min. The stained samples were then mounted with mounting medium (Polysciences, USA), and images were acquired via confocal microscopy. The fluorescence intensity was quantified via ImageJ, and the data were normalized to vehicle data.

#### Immunofluorescence staining

Eyecups were fixed in 4% paraformaldehyde at RT for 1 h and rinsed with PBS. They were then blocked with a solution containing 0.1% Triton X-100 and 3% BSA at RT for 1 h. Following blocking, the eyecups were incubated overnight at 4 °C with the following primary antibodies diluted in 1% BSA in PBS: anti-ZO-1 (Invitrogen, USA), anti-p16INK4A (Santa Cruz, USA), anti-p21 (Abcam, UK), and anti-HMGB1 (Abcam, UK). On the following day, appropriate secondary antibodies were added, and the samples were incubated at RT for 1 h. The eyecups were stained with Hoechst 33342 in PBS for 15 min at RT. Finally, the slides were mounted with mounting medium. Images were subjected to quantitative fluorescence analysis via confocal microscopy with Z-stacks and quantified with ImageJ.

To quantify ZO-1, which is localized at the RPE cell border outlining the shape of RPE cells, the area size of each sample was measured via the region of interest (ROI) in ImageJ. To highlight the ZO-1 areas, the region was painted with color proportional to their size, creating a pseudocolor representation, using the brightness/contrast adjuster (BAR) plugin in Fiji.

To quantify the other fluorescein stains, confocal images were captured in 4 random regions around the damaged area from each sample. The number of p16/p21/HMGB1-positive cells was determined via ImageJ software, and the proportion of each region to the entire flat mount was calculated.

#### Electroretinogram

Overall retinal function and visual defects were assessed via dark-adapted scotopic electroretinography (ERG). Three days after N201-gal injection, the mice were dark-adapted overnight and maintained under dim red light on a warming plate to regulate body temperature during the procedure. Following anesthesia and pupil dilation, a drop of 2% hyperellosing gel was applied directly to each eye to improve electrode contact and reduce discomfort. The scotopic ERGs were then recorded via a Celeris Diagnosys system (Diagnosys, USA) with white light stimuli (630 nm red, 515 nm green, and 455 nm blue) presented under dim red light. The electrodes were placed in a “touch–touch” configuration, with the stimulator positioned directly on the cornea. The ERGs were recorded from each eye across a range of intensities (0.001 to 10 cd s/m2), and 3 trials were averaged for each intensity step.

#### Statistical analysis

Statistical analysis was performed via a *t* test (unpaired, 2-tailed) or one-way analysis of variance (ANOVA) with Tukey’s multiple comparison post hoc test in GraphPad Prism (GraphPad Software, USA). All experimental results are expressed as the mean ± SEM, and *P* < 0.05 was considered statistically significant.

## Results and Discussion

### Synthesis and stability of N201-gal

**Fig. 2.**
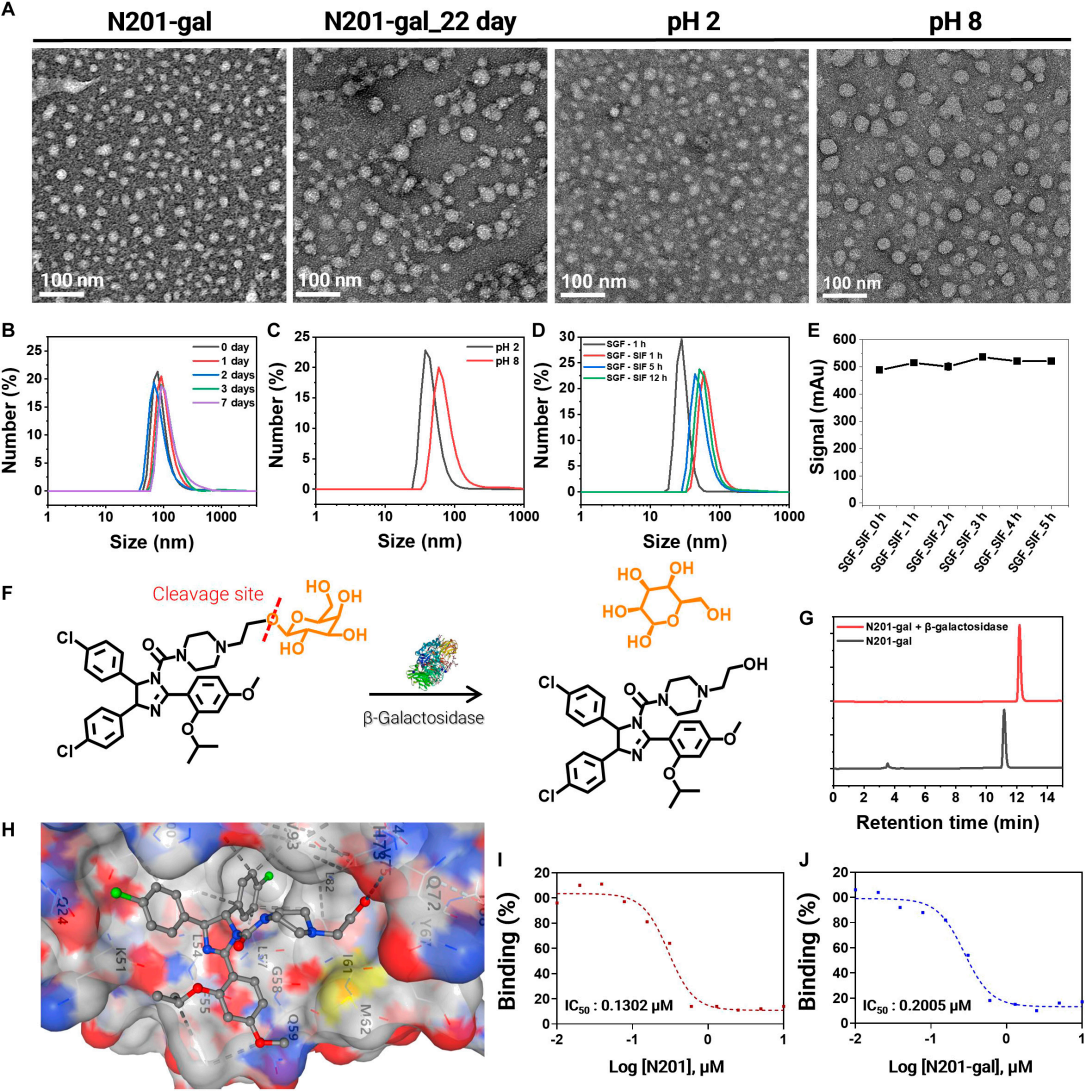
Fabrication of N201-gal, stable, and inhibitory MDM2 proteins. (A) Transmission electron microscopy (TEM) image showing that the stability of N201-gal depends on day and pH. (B) N201-gal diameter does not change during the 22nd day in water, 100 μM. (C) The pH changed from acidic to neutral, and the diameter slightly increased from 20 nm to 50 nm. (D) In the simulated gastric fluid and intestinal fluid, N201-gal was stable, and the diameter ranged from 20 nm to 50 nm. (E) High-performance liquid chromatography (HPLC) analysis of the stability of N201-gal at the 10.5-min peak. (F) Scheme of N201-gal cleavage to N201 by the β-galactosidase enzyme. (G) β-Galactosidase cleaves N201-gal into N201, which is present in 2 equilibriums of the enzyme. (H) Simulation of the interaction of N201 with MDM2. (I) Fluorescence polarization (FP) analysis of the interaction of N201 with MDM2 instead of with fluorescein isothiocyanate (FITC)–p53. The IC_50_ was 0.1302 μM. (J) FP analysis of N201-gal interacting with MDM2 instead of with FITC-p53. The IC_50_ was 0.2005 μM.

N201-gal, a novel compound designed for oral administration to target cellular senescence, with potential relevance to age-related retinal degeneration, represents a significant advancement in senolytic drug development. This compound combines the MDM2-inhibiting properties of Nutlin with the enhanced gastrointestinal absorption facilitated by galactose conjugation [19]. The synthesis of N201-gal involves a multistep process (Scheme S1). Initially, N201 is synthesized via the conjugation of the ethyl hydroxy piperazine. This intermediate is then further modified through a reaction with 2,3,4,6-tetra-*O*-acetyl-α-d-galactopyranosyl bromide via a silver-based catalyst, resulting in N201-gal-Ac. The final product, N201-gal, was obtained after the reaction under ammonium water and purification via liquid chromatography (LC) column chromatography (Figs. S1 to S6). The unique structure of N201-gal, featuring both hydrophobic (N201) and hydrophilic (galactose) moieties, enables it to maintain a stable spherical nanoparticle structure in PBS (Fig. 2A). CAC used to determine the minimum concentration required to maintain particle morphology was determined to be above 10 μM, ensuring particle formation at therapeutically relevant concentrations (Fig. S7).

Extensive stability testing has demonstrated the robustness of N201-gal under various conditions. The compound maintains its nanoparticle structure, with diameters ranging from 2 to 30 nm and a spherical shape, for extended periods (up to 22 d) when stored in aqueous solution (Fig. 2A and B). Additionally, dynamic light scattering (DLS) analysis confirmed that N201-gal maintained its nanoscale size distribution even in simulated gastric and intestinal fluids (Fig. S8). Furthermore, HPLC analysis revealed that N201-gal remained chemically stable in 55% serum for over 24 h (Fig. S9), supporting its suitability for systemic circulation and sustained release in vivo. Furthermore, N201-gal is stable across a range of pH conditions (pH 2 to 8), mimicking the environment encountered during gastrointestinal transit (Fig. 2C) [14]. This pH stability is particularly important for oral administration, as it suggests that the compound can withstand the acidic conditions of the stomach and the more neutral environment of the intestines. The stability of N201-gal in artificial gastric and intestinal fluids [20] further supports its potential as an oral medication [21]. While slight size increases were observed in the intestinal fluid, the overall integrity and spherical morphology of the nanoparticles were maintained (Fig. 2D). HPLC analysis confirmed the chemical stability of N201-gal in these artificial fluids over time (Fig. 2E). Importantly, N201-gal also demonstrated stability in blood serum (55% FBS in PBS), as verified by HPLC. This serum stability is crucial for maintaining the integrity of a compound after intestinal absorption and during systemic circulation, ensuring that it can reach its target tissues, such as the retina, in its active form. The comprehensive stability profile of N201-gal, encompassing various physiological conditions, strongly supports its potential as an orally administered senolytic drug. Its ability to maintain structural and chemical integrity throughout the gastrointestinal tract and in the bloodstream suggests that N201-gal could effectively reach SnCs in the retina, supporting a noninvasive senolytic approach for senescence-associated retinal degeneration.

### Uncapping of N201-gal by β-galactosidase and its interaction with MDM2

The drug is designed to leverage the overexpression of β-galactosidase in SnCs, and this overexpression serves as a biological trigger for drug activation. When N201-gal encounters these SnCs, β-galactosidase cleaves the galactose moiety, releasing the active N201 compound. This cleavage process is crucial for the drug’s selective action, ensuring that it primarily affects SnCs while minimizing its impact on healthy tissues. We confirmed galactose cleavage from N201-gal via the overexpression of β-galactosidase enzymes in senescent RPE cells (Fig. 2F and G) [22]. To further validate this activation mechanism, we conducted an enzymatic cleavage assay, which revealed a significant shift in the HPLC retention time upon β-galactosidase treatment, confirming the enzymatic release of N201 (Fig. S10). This selective activation ensures that N201-gal remains inactive in normal cells, reducing potential off-target effects. HPLC analysis provided clear evidence of the cleavage process, revealing a distinct shift in elution time from 11 to 12 min when N201-gal was exposed to β-galactosidase. This shift corresponds to the conversion of N201-gal to N201, confirming the successful activation of the drug. This cleavage mechanism was further validated via nuclear magnetic resonance (NMR) spectroscopy. The NMR data revealed specific shifts in the peaks associated with the galactose moiety and the hydroxyl group of the N201 component, providing molecular-level confirmation of the cleavage process.

Once cleaved, the resulting N201 molecule has a high affinity for binding to the MDM2 protein, which is crucial for its therapeutic effect. Molecular docking simulations elucidated the nature of this interaction, highlighting the importance of the benzene and hydroxyl groups of N201 in facilitating strong binding to MDM2 (Fig. 2H). This binding can lead to the overexpression of the p53 protein, a key factor in inducing apoptosis in SnCs. The binding efficacy of N201-gal and its cleaved form, N201, was quantitatively assessed via FP analysis. Interestingly, N201-gal had a binding concentration of 0.200 μM, whereas cleaved N201 exhibited a 1.5-fold greater binding at 0.130 μM (Fig. 2I and J). This difference suggests that the cleavage process not only activates the drug but also enhances its binding affinity to MDM2 (Figs. S11 and S12 and Table S1).

### Senolytic effect on the senescent ARPE-19 cell line

To further elucidate the senolytic properties of N201-gal, we conducted a series of experiments using the ARPE-19 cell line, a well-established model for retinal pigment epithelium studies. Although ARPE-19 cells lack certain physiological features of primary RPE cells, they have been extensively utilized as an in vitro model to study oxidative stress-induced damage and senescence-related pathways in retinal research. Although cellular senescence may contribute to AMD-related degeneration, not synonymous with aging or the disease itself, we focus on selective targeting of SnCs rather than modeling the full complexity of AMD or systemic aging. Cellular senescence was induced by doxorubicin (DOX) treatment [23] for 10 d, a method known to induce cellular senescence. While the DOX-induced senescence model in ARPE-19 cells does not replicate all features of AMD pathology, it offers a well-controlled and reproducible system to investigate senescence-associated stress responses in RPE cells. This approach allows for the evaluation of senolytic drug selectivity and mechanism of action under defined conditions [16]. The senescent state of the treated cells was confirmed through multiple complementary techniques. First, we employed the SA-β-gal assay [24], a widely recognized biomarker for cellular senescence. The X-galactosidase (X-gal) assay [25], visualized through confocal microscopy, revealed an increased intensity of green coloration in SnCs compared with that in normal cells. This enhanced β-galactosidase activity is a hallmark of senescence, confirming the effectiveness of our senescence induction protocol. To further validate the senescent phenotype, we examined the expression levels of p53 and p21, 2 key proteins involved in senescence pathways. Using immunofluorescence and confocal microscopy, we observed significantly elevated levels of both p53 and p21 in SnCs compared with their normal counterparts (Fig. S13). This up-regulation of senescence-associated proteins provided additional confirmation of the successful induction of senescence in our cell model.

With the establishment of the SnC model, we investigated the subcellular localization and mechanism of action of N201-gal. Our hypothesis was that N201-gal nanoparticles would enter SnCs and be cleaved by β-galactosidase, which is known to be overexpressed in the lysosomes of SnCs. To test this hypothesis, we developed an experimental approach using fluorescence tracking. We loaded N201-gal nanoparticles with IR780, a fluorescent dye, to create IR780@N201-gal nanoparticles. These labeled nanoparticles were purified through dialysis to remove any unencapsulated dye. SnCs were then treated with these fluorescent nanoparticles for 2 h, followed by washing and subsequent staining with a lysosome-specific tracker. Confocal microscopy analysis of these treated cells revealed strong colocalization between the IR780@N201-gal nanoparticles and the lysosomal tracker (Figs. S14 and S15). This overlap provides compelling evidence that N201-gal nanoparticles indeed localize to lysosomes within SnCs, positioning them in close proximity to the overexpressed β-galactosidase enzymes. These results collectively support our proposed mechanism of action for N201-gal. The nanoparticles effectively enter SnCs and accumulate in lysosomes, where they can interact with elevated levels of β-galactosidase. This interaction is crucial for the cleavage of N201-gal, which releases the active N201 compound and initiates its senolytic effects.

**Fig. 3.**
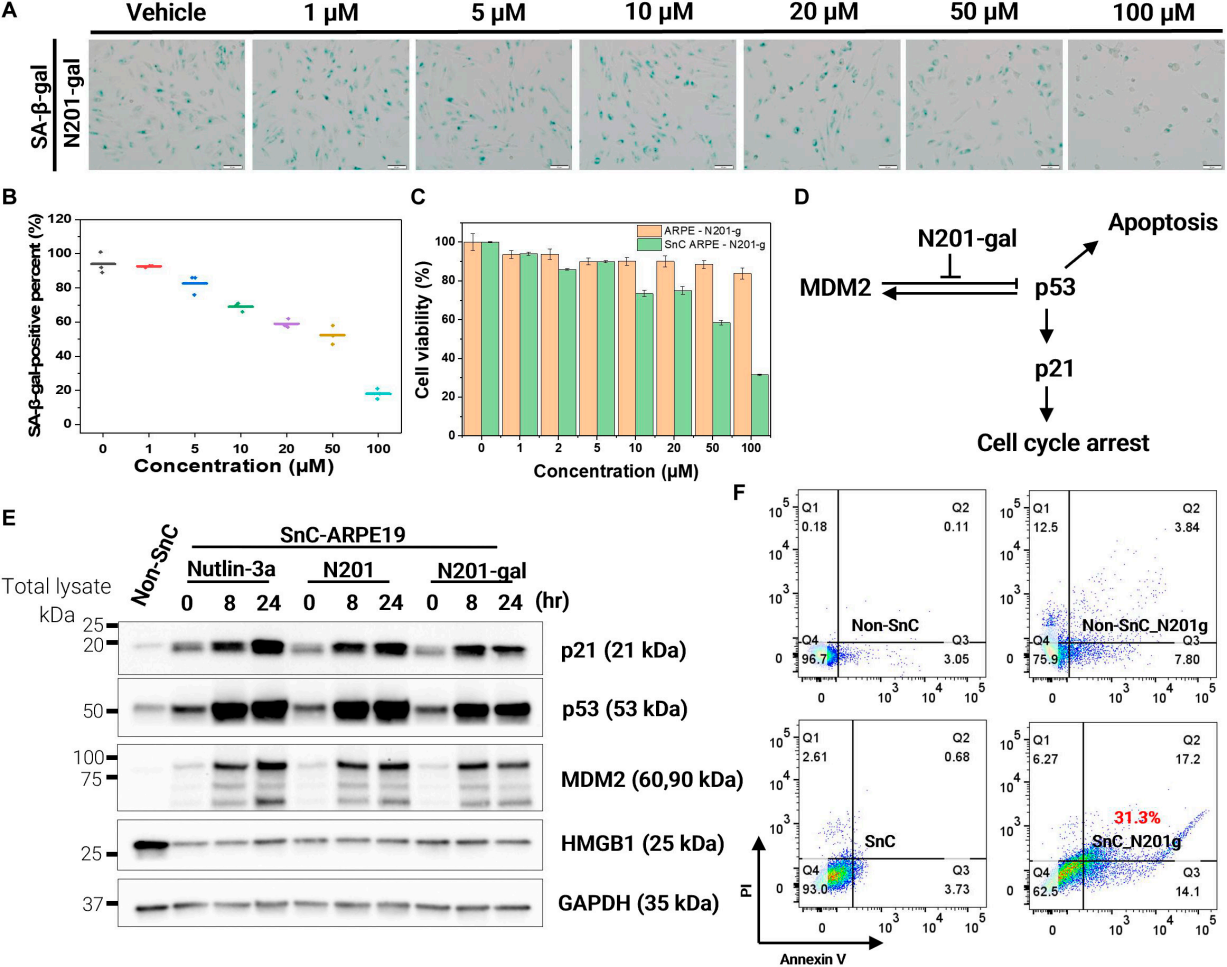
Selectively killing senescent ARPE19 cells. (A) X-galactosidase (X-gal) assay of N201-gal concentration-dependent effects. The green color indicates cleavage of X-gal by the β-galactosidase enzyme. (B) Graph of the percentage of senescence-associated β-galactosidase (SA-β-gal)-positive N201-gal cells. (C) Cell viability of N201-gal in the normal ARPE-19 cell line and the senescent ARPE-19 cell line. (D) N201-gal-mediated inhibition of MDM2 and overexpression of the p53 protein induce cell apoptosis. (E) Western blot of the Nutlin series over time; proteins associated with senescence and apoptosis are increased. (F) Flow cytometry measurements of cell apoptosis in ARPE-19 cell-derived non-SnCs and SnCs in the absence or presence of N201-gal (100 μM, 24 h) were performed via an annexin V-FITC/propidium iodide (PI) kit.

N201-gal, a novel senolytic compound, has a remarkable balance of efficacy and selectivity in targeting SnCs, representing a significant improvement over its predecessors, Nutlin-3a and N201. Initial MTT assays revealed that while all 3 compounds were highly effective at eliminating SnCs at 100 μM, N201-gal exhibited a unique profile of reduced overall toxicity while maintaining selectivity for SnCs (Fig. 3A and Figs. S16 to S18). Further analysis via flow cytometry confirmed that N201-gal selectively induced apoptosis in senescent ARPE-19 cells while sparing normal cells. Compared with Nutlin-3a and N201, N201-gal demonstrated significantly lower toxicity in non-SnCs, as evidenced by the reduced proportion of annexin V/PI-positive apoptotic cells. These findings highlight the improved therapeutic window of N201-gal over conventional MDM2 inhibitors. This selective action was visually confirmed by a marked decrease in SA-β-gal-positive cells following N201-gal treatment. Further quantitative analysis provided crucial insights into the relative potencies of the compounds. The IC_50_ values for Nutlin-3a and N201 were approximately 20 μM, indicating high potency. In contrast, N201-gal had a higher IC_50_ of approximately 50 μM (Fig. 3B). However, this apparent reduction in potency must be interpreted in the context of selectivity. When the effects on normal versus SnCs were compared, N201-gal demonstrated superior selectivity, a critical feature lacking in both Nutlin-3a and N201 (Fig. 3C). This selectivity is paramount in the development of safe and effective senolytic therapies, as it minimizes potential off-target effects on healthy tissues.

The molecular mechanism underlying the selective action of N201-gal was elucidated through detailed Western blot analyses. These studies revealed that the efficacy of N201-gal stems from its ability to disrupt the MDM2–p53 interaction, specifically in SnCs. This disruption initiates a cascade of molecular events leading to SnC death. The inhibition of MDM2 results in p53 protein overexpression, and elevated p53 levels subsequently lead to increased p21 protein expression. The overexpression of p21 induces cell cycle arrest, ultimately triggering apoptosis in SnCs (Fig. 3D). Time-course and concentration-dependent Western blot analyses at sublethal concentrations (20 μM) provided further insights into this mechanism. In normal cells, the baseline levels of p21, p53, and MDM2 were consistently low. SnCs, however, presented elevated baseline levels of p21 and p53 [24]. Upon N201-gal treatment, a rapid increase in p53 levels was observed within 8 h, followed by the subsequent up-regulation of p21 (Fig. 3E). Dose-dependent increases in both p53 and p21 were observed over a 24-h period, which strongly correlated with the induction of cell death.

To confirm that N201-gal induced apoptosis, we performed an annexin V-PI assay. In normal and senescent cells without drug administration, more than 90% of the cells remained alive. Nutlin-3a induced apoptosis in 48.5% of SnCs and 18% in normal cells, whereas N201 affected 40% of SnCs and 17.9% of normal cells. N201-gal, however, showed remarkable selectivity, inducing apoptosis in 17.2% of SnCs but only 11.64% of normal cells (Fig. 3F and Fig. S19). This marked difference in apoptotic induction between senescent and normal cells underscores the potential of N201-gal as a safe and effective senolytic agent. These findings were further corroborated by confocal microscopy via annexin V and PI staining. The results visually demonstrated widespread cell death (indicated by PI-positive staining) in SnCs treated with N201-gal at concentrations of 50 μM or higher, whereas normal cells remained largely unaffected (Fig. S20). Thus, N201-gal has emerged as a promising senolytic agent, demonstrating enhanced selectivity for SnCs compared with its precursors.

### Energy-dependent transport of N201-gal across the intestinal epithelium

**Fig. 4.**
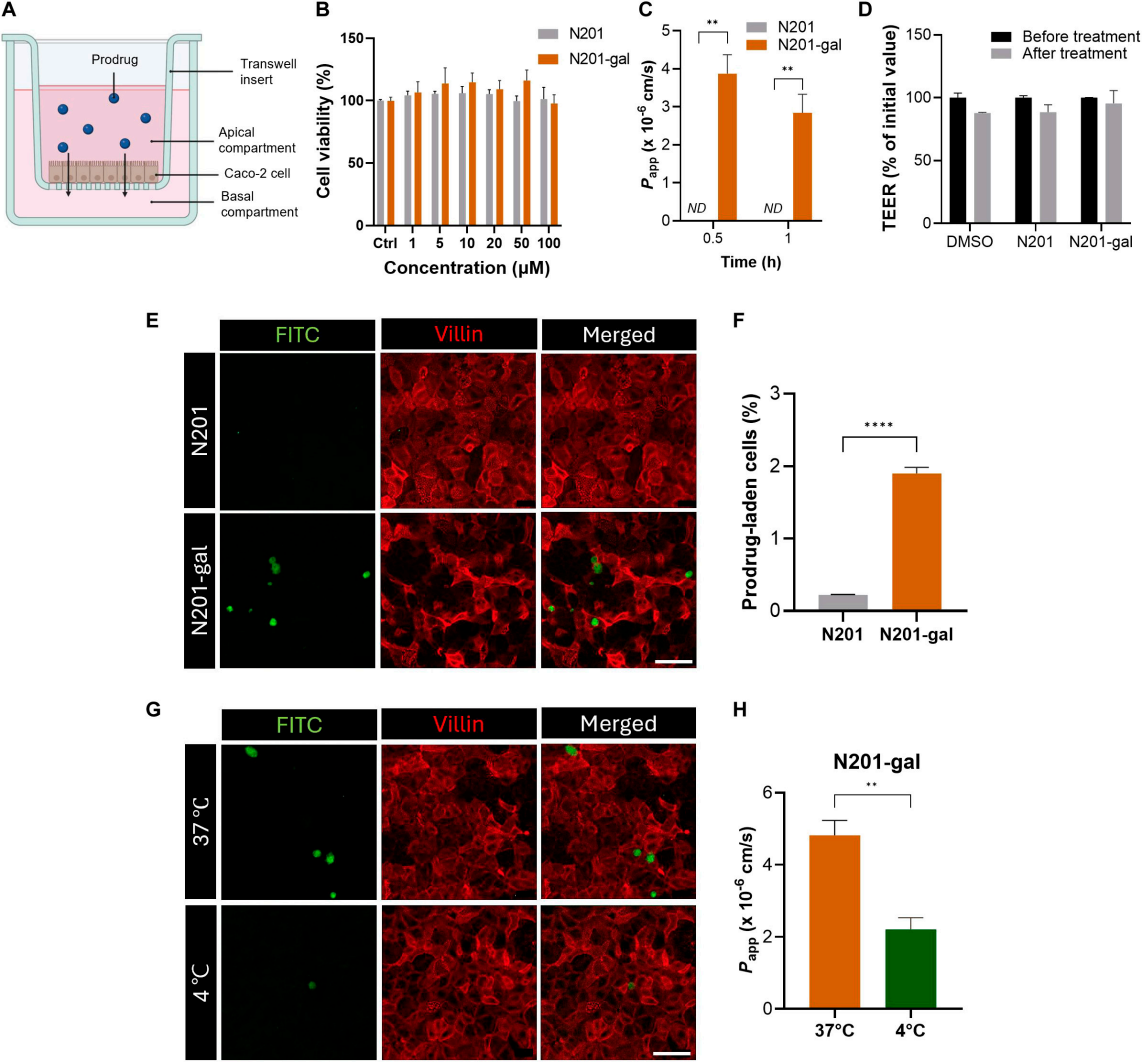
Transport of N201-gal across the Caco-2 monolayer. (A) Schematic illustration of the Caco-2 cell-based human intestine permeability model using a transwell system. (B) Cellular viability assay of 2D-cultured Caco-2 cells treated with various concentrations of N201 and N201-gal. (C) Apparent permeability (*P*_app_) of N201 and N201-gal across the Caco-2 monolayer. (D) Changes in the transepithelial electrical resistance (TEER) values of the Caco-2 transwell system after treatment with the prodrugs. (E) Immunofluorescence microscopic analysis of Caco-2 cells labeled with villin, an enterocyte marker, after 10-min treatments with N201 and N201-gal. (F) Ratio of prodrug uptake by enterocytes analyzed via ImageJ. (G) Microscopic immunofluorescence analysis of Caco-2 cells labeled with villin after treatment with N201-gal at 37 and 4 °C. (H) *P*_app_ values of N201-gal at 37 and 4 °C.

To assess the potential of N201-gal as an orally administered senolytic agent, we conducted a series of experiments to evaluate its ability to penetrate the intestinal epithelium. We employed a Caco-2 cell permeability model based on a transwell system [26], a well-established method for simulating intestinal absorption (Fig. 4A) [27]. Initially, we assessed the cytotoxicity of N201 and N201-gal on Caco-2 cells and found no cytotoxic effects at concentrations ranging from 1 to 100 μM (Fig. 4B). The penetration ability of the prodrugs was examined via the use of 10 μM FITC-labeled N201 and N201-gal (Scheme S2). These were applied to Caco-2 monolayers in the apical compartments of the transwell system, using HBSS buffer as the medium [28]. The integrity of the cell monolayer was confirmed by measuring TEER [29], with experiments proceeding when TEER values exceeded 300 Ω·cm^2^, typically achieved 6 d after cell seeding. N201-gal demonstrated moderate intestinal permeability, with a permeability coefficient (*P*_app_) of 3.36 ± 0.82 × 10^−6^ cm/s. In contrast, N201 was not detected in the basal compartment, indicating significantly lower permeability (Fig. 4C). Notably, there was no significant change in TEER during the permeability assay, suggesting that the transport of N201-gal did not compromise intestinal barrier integrity (Fig. 4D).

Immunofluorescence microscopic analysis revealed significantly greater cellular uptake of FITC-labeled N201-gal in villin-labeled enterocytes, which absorb molecules from the gut lumen in the small and large intestines [30], than did FITC-labeled N201 (Fig. 4E). The uptake percentage of N201-gal by enterocytes was 8.5 times higher than that of N201 (Fig. 4F). To further investigate the transport mechanism of N201-gal, we examined the change in permeability at low temperature conditions (4 °C), which are known to suppress active transport processes, including endocytosis [31]. The Caco-2 cell monolayers were preincubated for 1 h at 4 and 37 °C to achieve thermal equilibrium and then treated with N201-gal for 30 min at 4 and 37 °C, respectively. Immunofluorescence images revealed a significant reduction in N201-gal cellular uptake at 4 °C compared to 37 °C (Fig. 4G). Consequently, the transport of N201-gal across the Caco-2 monolayer was markedly decreased at 4 °C (Fig. 4H). These results suggest that the cellular uptake of N201-gal is mediated via an active mechanism, rather than passive diffusion. Overall, these findings provide strong evidence for the superior intestinal permeability and cellular uptake of N201-gal compared to N201. This enhanced permeability, coupled with the absence of cytotoxicity and maintenance of barrier integrity, positions N201-gal as a promising candidate for oral administration in senolytic therapy.

### Intravitreal injection of N201-gal eliminates senescent RPE cells in an in vivo DOX-induced retinal senescence model

In a previous study, we demonstrated that the potential therapeutic effects of Nutlin-3a in selectively eliminating senescent RPE cells protect against age-related vision loss by supporting RPE cell function and ameliorating AMD-like pathology in a mouse model [32]. AMD, one of the leading causes of visual impairment in elderly individuals, is characterized by progressive degeneration of the RPE and photoreceptor cells [33], ultimately leading to vision impairment and blindness. The accumulation of SnCs plays a crucial role in the aging process and the development of age-related diseases such as AMD. While SnCs typically perform various physiological roles in maintaining homeostasis and are eliminated by the immune system [34], age-related immune system degeneration and decreased self-healing ability lead to their accumulation, resulting in chronic inflammation and an increased risk of various geriatric diseases [35].

**Fig. 5.**
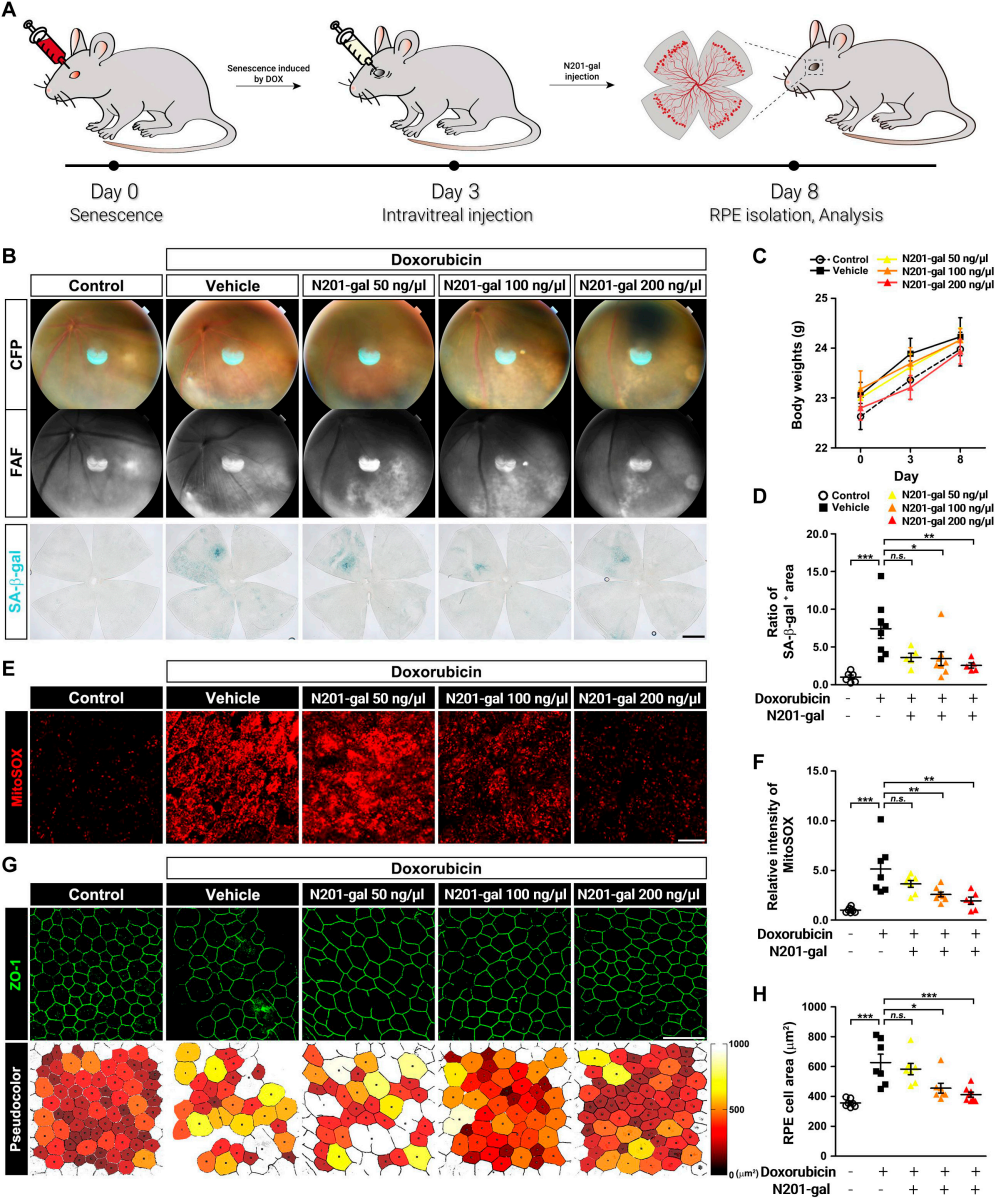
Doxorubicin-induced RPE senescence and reactive oxygen species (ROS) generation were diminished with N201-gal treatment. (A) Scheme of the animal experiment. (B) Representative images of fundus angiography [color fundus photography (CFP; top row)] and autofluorescence images (middle row). The SA-β-gal-positive cells are stained blue-green, with images showing the senescent area in the RPE (bottom row). Original magnification, ×4; scale bar, 200 μm. (C) Body weights of the mice in all 5 groups. (D) Quantification of β-galactosidase activity (control, *n* = 6; vehicle, *n* = 8; N201-gal 50 ng/μl, *n* = 5; N201-gal 100 ng/μl, *n* = 8; N201-gal 200 ng/μl, *n* = 5; *P < 0.05, **P < 0.01, ***P < 0.001). (E) Mitochondrial superoxide levels in the RPE were detected via MitoSOX solution (red). Original magnification, 63×; scale bar, 20 μm. (F) Comparison of mitochondrial superoxide levels between the groups (control, *n* = 8; vehicle, *n* = 7; N201-gal 50 ng/μl, *n* = 7; N201-gal 100 ng/μl, *n* = 8; N201-gal 200 ng/μl, *n* = 6; **P < 0.01, ***P < 0.001). (G) Immunofluorescence analysis of zonula occludens-1 (ZO-1) (green, top row) and pseudocolor images (bottom row) showing the morphology and area of RPE tight junctions. Original magnification, ×40; scale bar, 50 μm. (H) Quantification of the cell area via region of interest (ROI) analysis (control, *n* = 7; vehicle, *n* = 7; N201-gal 50 ng/μl, *n* = 7; N201-gal 100 ng/μl, *n* = 7; N201-gal 200 ng/μl, *n* = 8; *P < 0.05, ***P < 0.001).

To investigate the effects of N201-gal on senescent RPE cells, we developed an AMD-like mouse model via the use of doxorubicin, a DNA-damaging agent known to induce rapid senescence in RPE cells [8]. Our results demonstrated that N201-gal treatment significantly reduced AMD-like pathogenesis in a dose-dependent manner. Briefly, we injected 100 ng/μl doxorubicin into the subretinal space and then intravitreally injected N201-gal or vehicle 3 d later (Fig. 5A). There were no significant changes in body weight during the experimental period (Fig. 5C). Loss of the RPE and subsequent photoreceptor loss are observed in the DOX-induced retinal senescence model [36]. When atrophy of the RPE becomes severe, geographic atrophy (GA) occurs, and cell loss causes vision to deteriorate [37]. We used CFP and autofluorescence imaging (FAF) to determine whether N201-gal reduced advanced AMD-like pathogenesis [38]. Fundoscopy revealed that doxorubicin induces GA throughout the eye, but N201-gal treatment significantly reduced GA in a dose-dependent manner. Lipofuscin, which is thought to be an incompletely degraded product that accumulates within RPE cells [39], was detected via fundus autofluorescence imaging. We observed a decrease in lipofuscin, which appeared as dark and hypofluorescent regions, in a dose-dependent manner in response to N201-gal treatment. Additionally, the activity of SA-β-gal, one of the most important senescence markers, was significantly decreased in response to 100 or 200 ng/μl N201-gal treatment, indicating that N201-gal eliminated senescent RPE cells (Fig. 5B and D).

Oxidative stress is known to be a cause of AMD and mainly causes macular degeneration by damaging the RPE and choroid capillary. Damage to the RPE is induced by lipofuscin deposition, resulting in the generation of ROS [40]. Superoxide production in live cells was measured via MitoSOX Red, which can infiltrate live cells and selectively target mitochondria. The results revealed that the increase in ROS caused by doxorubicin was diminished with N201-gal treatment, especially the fluorescence signal at 200 ng/μl N201-gal, which decreased to almost the background level. Therefore, the fluorescence intensity revealed that the level of ROS decreased with N201-gal treatment (Fig. 5E and F). Moreover, unlike the higher dose of N201-gal, 50 ng/μl of N201-gal did not significantly change.

The RPE is a hexagonal single-cell layer lying on the inner surface of Bruch’s membrane that plays an important role in visual function through interactions with surrounding tissue, photoreceptors, and the choriocapillaris [41]. It has tight junctions, which are tightly connected and become enlarged, irregularly shaped, and disrupted with senescence [42]. The localization of zonula occludens-1 (ZO-1), one of the most widely known tight junction proteins, was visualized via immunohistochemistry [43]. Treatment with N201-gal restored the senescent RPE, as confirmed by pseudocolor images (Fig. 5G). There was no significant difference in the effects of N201-gal treatment at 50 ng/μl, but compared with doxorubicin treatment alone, N201-gal treatment appeared to restore disrupted RPE tight junctions (Fig. 5H).

Therefore, intravitreal injection of N201-gal resulted in the elimination of senescent RPE cells in the DOX-induced retinal senescence model. This was evidenced by the significant reduction in GA and lipofuscin accumulation, both of which are hallmarks of AMD progression [17]. Additionally, N201-gal treatment decreased the activity of SA-β-gal, indicating a reduction in cellular senescence. Moreover, N201-gal treatment attenuated oxidative stress, as evidenced by reduced ROS production, particularly at relatively high concentrations. These findings suggest that N201-gal exerts its protective effects by targeting multiple pathways involved in senescence-associated retinal pathology, including those involved in senescence and oxidative stress. Furthermore, N201-gal treatment restored the integrity of tight junctions in the RPE layer, as indicated by the localization of ZO-1. This restoration of tight junctions is crucial for maintaining the barrier function of the RPE and preserving retinal homeostasis.

**Fig. 6.**
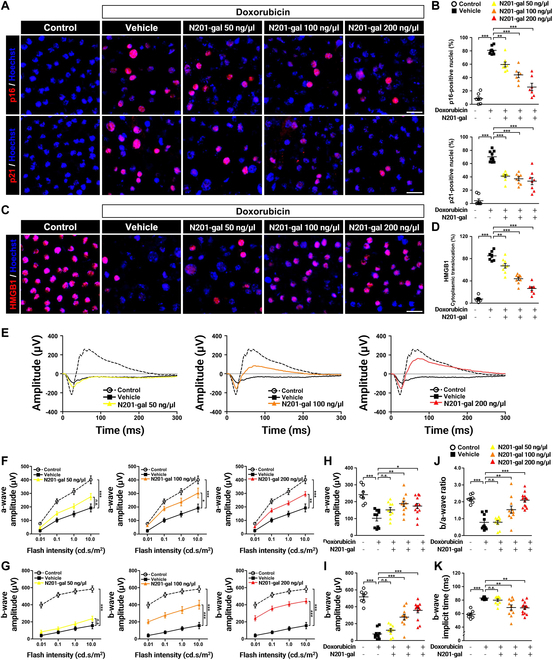
N201-gal eliminated SnCs and restored fundus functions. (A) Images of major markers of cellular senescence, namely, p16 (top row) and p21 (bottom row), and (C) the inflammatory factor HMGB1; the nuclei were counterstained with Hoechst (blue). Original magnification, 63×; scale bar, 20 μm. The ratio of (B) p16- and p21-positive cells or (D) HMGB1 cytoplasmic translocation (p16; control, *n* = 7; vehicle, *n* = 8; N201-gal 50 ng/μl, *n* = 8, N201-gal 100 ng/μl, *n* = 7; N201-gal 200 ng/μl, *n* = 7; p21; control, *n* = 8; vehicle, *n* = 10; N201-gal 50 ng/μl, *n* = 9, N201-gal 100 ng/μl, *n* = 8; N201-gal 200 ng/μl, *n* = 8; HMGB1; control, *n* = 8; vehicle, *n* = 7; N201-gal 50 ng/μl, *n* = 9, N201-gal 100 ng/μl, *n* = 9; N201-gal 200 ng/μl, *n* = 8; **P < 0.01, ***P < 0.001). (E) Images of the mean electroretinography (ERG) waveforms for the 5 groups of mice obtained under dark-adapted conditions and generated via 0.1 cd s/m^2^ flashes are presented. The line graph shows (F) a-wave and (G) b-wave amplitudes in response to a series of single light flashes with intensities ranging from 0.01 to 10 cd s/m^2^. Quantitative evaluation of the amplitudes at a flash intensity of 0.1 cd s/m^2^ for (H) a-waves and (I) b-waves. (J) Average plot of the b/a wave ratio for the control and the 4 treatment groups. In response to light flashes of 0.1 cd s/m^2^, (K) the b-wave implicit time was measured as the time difference between flash onset and peak. This time delay was improved with high-dose N201-gal treatment but was no longer statistically significant in the 50 ng/μl N201-gal group (control, *n* = 7; vehicle, *n* = 10; N201-gal 50 ng/μl, *n* = 10; N201-gal 100 ng/μl, *n* = 11; N201-gal 200 ng/μl, *n* = 13; *P < 0.05, **P < 0.01, ***P < 0.001).

Among the various specific markers that are expressed in SnCs, p16 and p21 are classical senescence-related positive markers [44]. As shown in Fig. 6A and B, doxorubicin significantly up-regulated p16 and p21, and their expression levels strongly decreased with N201-gal treatment. These findings suggest that N201-gal treatment restrained RPE senescence. We next examined whether inflammatory substances, such as cytosolic HMGB1, which are attracting attention as aging-inducing factors, are decreased by N201-gal treatment. HMGB1 is normally localized in the nucleus, where it stabilizes nucleosomes and regulates the transcription of many genes. In contrast, extracellular HMGB1 is a mediator of danger-associated molecular patterns (DAMPs) [45]. Although we did not measure extracellular HMGB1 levels, it was assumed to be secreted in the absence of double staining in the nucleus. To evaluate the effect of N201-gal on the translocation of HMGB1 in the RPE, we performed immunostaining and compared the changes. As a result, HMGB1 was decreased by doxorubicin treatment, but N201-gal increased HMGB1 translocation to the nucleus (Fig. 6C and D).

To determine the effectiveness of N201-gal in restoring retinal function, we assessed the functional integrity of the retina via ERG. ERG was performed on dark-adapted mice, and the eyes were exposed to light flashes of sequentially increasing intensities. Images of the mean ERG waveforms for the 5 groups of mice obtained under dark-adapted conditions and generated via 0.1 cd s/m^2^ flashes are presented in Fig. 6E. Treatment with 200 ng/μl N201-gal (red, right) resulted in almost complete recovery to the same level as the control (black, dashed line), and 100 ng/μl N201-gal (orange, middle) slightly decreased in amplitude. However, treatment with 50 ng/μl N201-gal (yellow, left) resulted in a significant reduction, similar to the response to doxorubicin treatment alone (black, straight line). The a-wave, the negative trough at the beginning of the graph, represents mainly photoreceptor function, whereas the b-wave, which is measured from the a-wave trough to the peak of the wave, is indicative of the response predominantly from bipolar cells. Compared with those in the control group (white), the a-wave and b-wave amplitudes were significantly lower in the doxorubicin-treated group (black) (Fig. 6F and G). This finding indicated the presence of photoreceptor and bipolar cell function defects. Treatment with 50 ng/μl N201-gal (yellow) had no effect or had a limited effect on the ERG response, whereas increasing the N201-gal concentration resulted in a statistically improved response at all flash points (from 0.01 to 10 cd s/m^2^). At the 0.1 cd s/m^2^ flashpoint, N201-gal recovered a-wave and b-wave amplitudes, as shown in Fig. 6H to K. Specifically, the amplitudes with 100 ng/μl N201-gal (orange) and 200 ng/μl N201-gal (red) were close to those of the vehicle. These findings suggest that N201-gal eliminates SnCs and restores RPE atrophy and the secondary loss of photoreceptor cells.

As a result, N201-gal treatment down-regulated the expression of the senescence markers p16 and p21, further supporting its ability to inhibit RPE senescence. Interestingly, N201-gal treatment also modulated the translocation of high-mobility group box 1 (HMGB1), a mediator of inflammation and aging. While doxorubicin treatment reduced HMGB1 levels, N201-gal promoted its translocation to the nucleus, suggesting a potential anti-inflammatory effect. This modulation of HMGB1 may contribute to the overall reduction in inflammation and senescence observed with N201-gal treatment. Assessment of retinal function via ERG revealed that N201-gal treatment improved both photoreceptor and bipolar cell function, indicating the restoration of retinal function. These functional improvements were dose dependent, with higher concentrations of N201-gal resulting in greater restoration of ERG responses. These findings underscore the therapeutic potential of N201-gal in not only eliminating SnCs but also promoting functional recovery of the retina.

### Oral administration of N201-gal eliminates senescent RPE cells

**Fig. 7.**
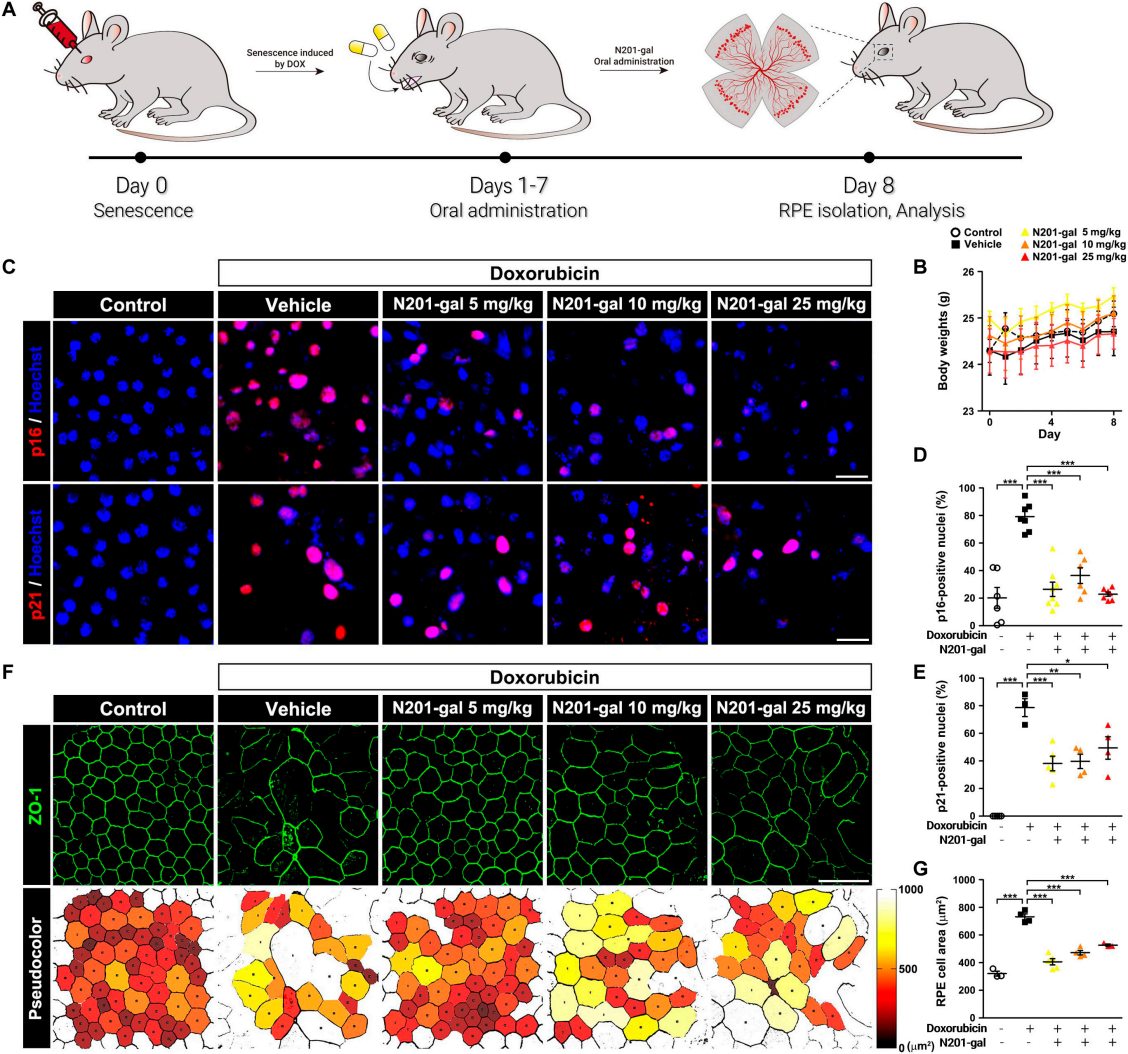
Oral administration of N201-gal reduced RPE senescence. (A) Scheme of the animal experiment. (B) Body weight changes in all 5 groups. (C) Images of the cellular senescence markers p16 (top row) and p21 (bottom row). Nuclei were counterstained with Hoechst (blue). Original magnification, 63×; scale bar, 20 μm. The ratios of (D) p16- and (E) p21-positive cells are shown in the graph and represent ameliorated RPE senescence following N201-gal treatment (p16; control, *n* = 6; vehicle, *n* = 7; N201-gal 5 mg/kg, *n* = 8; N201-gal 10 mg/kg, *n* = 6; N201-gal 25 mg/kg, *n* = 7; p21; control, *n* = 5; vehicle, *n* = 3; N201-gal 5 mg/kg, *n* = 5; N201-gal 10 mg/kg, *n* = 4; N201-gal 25 mg/kg, *n* = 4; *P < 0.05, **P < 0.01, ***P < 0.001). (F) Representative images of ZO-1 (green, top row) and pseudocolor images (bottom row). Original magnification, ×40; scale bar, 50 μm. Immunofluorescence images were quantified via ROI analysis and are shown in (G). The enlarged and disrupted tight junctions were recovered with 5 mg/kg N201-gal via oral gavage (control, *n* = 3; vehicle, *n* = 4; N201-gal 5 mg/kg, *n* = 5; N201-gal 10 mg/kg, *n* = 4; N201-gal 25 mg/kg, *n* = 3; ***P < 0.001).

We next sought to determine whether oral gavage of N201-gal could eliminate senescent RPE cells [46]. Oral administration was conducted via general mouse gavage, and the mice were continuously orally administered doxorubicin one time per day for 7 d after doxorubicin was injected to induce RPE senescence. There was no significant alteration in body weight after oral administration during the experimental observation period from day 0 to day 8 (Fig. 7B), suggesting that oral administration of N201-gal was well tolerated. This is an important finding for potential future clinical applications, as it indicates a favorable safety profile. Significantly fewer p16-positive cells were observed in all treatment groups, and the presence of p21, another senescence indicator, was also significantly lower (Fig. 7C to E). Additionally, substantial reductions in the RPE cell area were detected in all the injected groups (Fig. 7F and G), suggesting a potential optimal dosage range.

These findings demonstrate that orally administered N201-gal can be absorbed from the gastrointestinal tract, cross the blood–retina barrier, and reach the retina at concentrations sufficient to eliminate SnCs. In addition to intravitreal injection, oral administration of N201-gal also showed promising results in eliminating senescent RPE cells. Oral gavage of N201-gal led to a reduction in the senescent RPE cell area and eliminated SnCs, demonstrating the systemic absorption and bioavailability of N201-gal. These findings underscore the potential of N201-gal as a senolytic agent for retinal senescence, with possible translational relevance to AMD and other age-related retinal diseases. Furthermore, the ability to administer N201-gal orally offers a convenient and noninvasive treatment option for patients. These findings suggest that N201-gal could significantly impact clinical management strategies for senescence-associated retinal degeneration, providing an accessible and effective means of targeting senescent RPE cells and potentially slowing disease progression.

## Conclusion

N201-gal represents a significant advancement in the senolytic drug development, offering a noninvasive oral senolytic drug that specifically targets β-galactosidase in SnCs. This novel approach addresses a critical need in AMD therapy by providing a targeted, less invasive alternative to current treatments. N201-gal effectively targets and eliminates senescent retinal pigment epithelial (RPE) cells and alleviates senescence-associated retinal pathology in DOX-induced models. N201-gal inhibits the MDM2 protein through a β-galactosidase-induced mechanism, allowing for selective cleavage of its precursor in SnCs. This selectivity is crucial, as it leads to the release of an active agent that promotes p53 overexpression specifically in these cells. By exerting its effects on SnCs, N201-gal minimizes systemic side effects while maximizing its therapeutic impact on diseased tissues.

One of the key strengths of N201-gal is its stability under various physiological conditions, which is a critical factor for its efficacy as an oral medication. The study demonstrated improved intestinal absorption when galactose was included in the oral drug formulation, enhancing its bioavailability. This feature is particularly important for ensuring that the drug reaches its target cells at sufficient concentrations to exert its therapeutic effects. The efficacy of N201-gal was demonstrated through both intravitreal injection and oral administration in vivo. Both routes of administration led to significant reductions in cellular senescence indicators and oxidative stress in RPE cells. These effects are crucial for potentially slowing the progression of AMD, as SnCs and oxidative stress are key contributors to disease pathology. Furthermore, N201-gal treatment restored the integrity of RPE tight junctions. This is a critical finding, as the maintenance of these junctions is essential for preserving the retinal barrier and overall retinal function. By addressing this aspect of AMD pathology, N201-gal represents a comprehensive approach for treating this disease. The potential for systemic absorption and direct targeting of intraretinal SnCs through oral administration is perhaps one of the most promising aspects of N201-gal. This route of administration offers a more convenient and less invasive treatment option than existing intravitreal injections do, which are currently the standard of care for many retinal diseases. The ability to deliver effective treatment through oral medication could significantly improve patient compliance and quality of life, potentially leading to better long-term outcomes in AMD management.

In conclusion, N201-gal represents a promising new approach with potential relevance to senescence-associated retinal degeneration that combines targeted senolytic action with the convenience of oral administration. Given its enhanced bioavailability and selective activation in senescent RPE cells, N201-gal has strong potential for clinical translation. Importantly, its dual mode of administration (intravitreal and oral) provides flexibility in treatment strategies, potentially improving patient compliance and expanding therapeutic options. Future studies will focus on optimizing its pharmacokinetics and conducting clinical trials to validate its efficacy and safety for human use. Its mechanism of action, stability, and efficacy in reducing senescence and oxidative stress, along with its ability to restore retinal barrier function, position it as a potential platform in the field of retinal degenerative disease treatment. To further validate the therapeutic potential of N201-gal, future studies should consider incorporating chronic AMD models that better recapitulate hallmark features such as drusen formation, complement activation, and immune cell infiltration. Such models would enhance the translational relevance of senolytic approaches in AMD. Nonetheless, the present findings establish a valuable proof of concept for a β-galactosidase-responsive oral senolytic prodrug that selectively eliminates SnCs in RPE cells and offer a foundation for further translational development.

## Ethical Approval

The study does not involve human participants, human data, or tissues. All animal experiments were conducted following the guidelines of the IACUC of Konkuk University. The study was approved under the IACUC protocol number KU21118.

## Data Availability

No datasets were generated or analyzed during the current study.

## Supplementary Materials

Supplementary 1 Synthesis and methods NMR spectra Schemes S1 and S2 Figs. S1 to S20 Table S1
