# Supplementary material for: A Self-Assembling Senolytic Prodrug with Enhanced Bioavailability and Selective Activation for Targeting Senescent Retinal Pigment Epithelium
Source: Biomater Res. 2025 Oct 1;29:0261. doi: 10.34133/bmr.0261 (PMC12484856; doi:10.34133/bmr.0261)
Supplement: Supplementary 1 — Synthesis and methods NMR spectra Schemes S1 and S2 Figs. S1 to S20 Table S1 [file bmr.0261.f1.docx]

Supporting Information

**A Self-Assembling Senolytic Prodrug with Enhanced Bioavailability and Selective Activation for Targeting Senescent Retinal Pigment Epithelium**

Haewon Ok^‡,a^, Hyun-Seo Park^b^, Jungin Park^c^, Sunyoung Hwang^b^, Jiwon Jang^a^, Jiye Kim^b^, Gaeun Park^a^, Dojoon Park^d^, Tae-Eun Park*^c^, Chaekyu Kim*^,b^, and Ja-Hyoung Ryu*^,a^

Table of contents

1. Synthesis and methods
2. NMR spectra
3. Supplementary figures
4. Synthesis and methods

Synthesis of N201-gal

Supplementary scheme S1. The synthesis method of compound N201-gal.

Compound 2 (N201)

A solution of rel-(4R,5S)-4,5-bis(4-chlorophenyl)-2-(2-isopropoxy-4-methoxyphenyl)-4,5-dihydro-1H-imidazole (1 g, 2.19 mmol) was added to the triphosgene (630 mg, 2.19 mmol) in DCM, TLC is change into carbamoyl chloride form, 2-hydroxy ethanol (857 mg, 6.57 mmol) is added in the solution. The mixture extraction with water, dried over Na_2_SO_4_ and concentrated under reduced pressure. The crude was purified by flash chromatography on silica gel (MeOH:CH_2_Cl_2_ = 1:99 to 10:90) to yield compound 2 (1.3 g, 97%). ^1^H-NMR (400mHz, chloroform-d) δ 7.48 (d, J = 8.4 Hz, 1H), 7.08 – 7.05 (m, 2H), 7.04 – 6.99 (m, 2H), 6.95 – 6.91 (m, 2H), 6.86 – 6.81 (m, 2H), 6.54 – 6.48 (m, 2H), 5.58 (d, J = 9.9 Hz, 1H), 5.42 (d, J = 9.8 Hz, 1H), 4.60 (p, J = 6.1 Hz, 1H), 3.84 (s, 3H), 3.52 (s, 2H), 3.12 (s, 4H), 2.35 (t, J = 5.3 Hz, 2H), 2.07 (s, 4H), 1.37 (dd, J = 18.8, 6.0 Hz, 6H).

Compound 3 (N201-gal-Ac)

To compound 2 (2.08 g, 3.405 mmol), silver carbonate (258.1 mg, 0.936 mmol), and 2,3,4,6-Tetra-o-acetyl-alpha-d-galactopyranosyl bromide (700 mg, 1.702 mmol), 4Å molecular sieve (50 mg) dissolved in DCM (85 ml). Purged by argon, the reaction mixture was stirred RT for overnight and solvent removed. The solution was filtered through celite to remove solids and filtrate concentrated in vacuum. The residue was dissolved in DCM, extract with saturated NaHCO_3_, water and brine. The organic layer was dried over MgSO_4_, filtered and concentrated in vacuum. The crude was purified by flash chromatography on silica gel (MeOH:CH_2_Cl_2_ = 1:99 to 10:90) to yield compound 3 (1.3 g, 40%). 1H NMR (400 MHz, Chloroform-d) δ 7.47 (d, J = 8.4 Hz, 1H), 7.03 (dd, J = 21.2, 8.0 Hz, 5H), 6.93 (d, J = 8.1 Hz, 2H), 6.83 (d, J = 8.1 Hz, 2H), 6.55 – 6.47 (m, 2H), 5.58 (d, J = 9.8 Hz, 1H), 5.46 – 5.31 (m, 2H), 5.03 (dd, J = 10.8, 3.9 Hz, 1H), 4.64 – 4.44 (m, 2H), 4.16 – 4.07 (m, 2H), 3.83 (s, 3H), 3.51 (t, J = 5.3 Hz, 2H), 3.12 (t, J = 5.0 Hz, 4H), 2.35 (t, J = 5.3 Hz, 2H), 2.17 – 1.95 (m, 14H), 1.38 (d, J = 6.0 Hz, 3H), 1.34 (d, J = 6.0 Hz, 3H), 1.23 (t, J = 7.1 Hz, 2H).

Compound N201-gal

To a solution of compound 3 (1 g, 1.06 mmol) in MeOH, ammonium in water is added to a solution by excess. After TLC is changed into below compound 2, the solution concentrated under reduced pressure. The crude was dissolved in DCM again, purified by flash chromatography on (MeOH:CH_2_Cl_2_ = 1:99 to 10:90) to yield N201-gal (300 mg, 36%). ^1^H NMR (400 MHz, Methanol-d4) δ 7.54 – 7.50 (m, 1H), 7.12 (d, J = 7.7 Hz, 2H), 7.04 (dd, J = 19.5, 8.1 Hz, 4H), 6.91 (d, J = 7.9 Hz, 2H), 6.66 (s, 2H), 5.77 – 5.68 (m, 2H), 5.49 (d, J = 10.4 Hz, 1H), 4.71 (s, 1H), 4.16 (s, 1H), 3.88 – 3.85 (m, 3H), 3.81 (s, 1H), 3.75 – 3.65 (m, 3H), 3.53 (s, 2H), 3.17 (s, 4H), 2.38 (s, 2H), 2.07 (d, J = 40.6 Hz, 4H), 1.36 (dd, J = 17.8, 5.7 Hz, 6H).

Synthesis of FITC-N201-ser-gal

Supplementary scheme S2. The synthesis method of FITC-N201-gal.

Compound 4 (Fmoc-ser-OH)

Fmoc-L-Ser(t-Bu)-OH (1 g, 2.61 mmol) were treated with TFA/DCM 1:1 (30 mL, RT, 2 h) to selectively remove the tert-butyl group. After removal of TFA and DCM under nitrogen stream, the residue dissolved in DCM. The crude was dissolved in DCM again, purified by flash chromatography on (MeOH:CH_2_Cl_2_ = 1:99 to 10:90) to yield Compound 4 (800 mg, 80%).

Compound 5 (Fmoc-ser-gal)

beta-D-Galactose pentaacetate (2.73 g, 7 mmol) and Fmoc-L-ser-OH (2.89 g, 8.37 mmol) were dissolved in 70ml ACN and BF_3_Et_2_O (2.64 ml, 21 mmol) was added slowly under nitrogen atmosphere. After 1h stirring at room temperature the reaction mixture was diluted with DCM and washed with 1M HCl and H_2_O. the organic phase was dried with MgSO_4,_ and the solvents were evaporated. The crude was dissolved in DCM again, purified by flash chromatography on (MeOH:CH_2_Cl_2_ = 1:99 to 10:90) to yield compound 5 (2.3 g, 50%). ^1^H NMR (400 MHz, Chloroform-d) δ 7.76 (d, J = 7.5 Hz, 2H), 7.59 (s, 2H), 7.40 (t, J = 7.5 Hz, 2H), 7.31 (t, J = 7.4 Hz, 2H), 5.37 (d, J = 3.4 Hz, 1H), 4.98 (dd, J = 10.5, 3.5 Hz, 1H), 4.50 (s, 1H), 4.44 (d, J = 8.5 Hz, 2H), 4.26 (d, J = 15.5 Hz, 1H), 4.21 (d, J = 9.3 Hz, 1H), 4.19 – 4.14 (m, 1H), 4.07 (dd, J = 11.2, 6.5 Hz, 1H), 3.95 (d, J = 10.5 Hz, 1H), 3.82 (t, J = 6.4 Hz, 1H), 2.14 (s, 3H), 2.08 – 1.90 (m, 9H).

Compound 6 (Fmoc-N201-ser-gal-Ac)

A mixture of compound 5 (100 mg, 0.152 mmol), N201 (102.3 mg, 0.167 mmol), and DMAP (1.86 mg, 0.015 mmol) in DCM was stirred at 0℃. Then EDC (34.98 mg, 0.182 mmol) was added to the solution. The reaction mixture was stirred for 1h at 0℃ and then stirred overnight at room temperature. The reaction mixture was subsequently concentrated in vacuum, and dissolved with DCM, after which the solution was washed with 1M HCl and saturated NaHCO_3_, and brine. The organic phase was dried over MgSO_4_, filtered, and evaporated in vacuo. The crude was dissolved in DCM again, purified by flash chromatography on (MeOH:CH_2_Cl_2_ = 1:99 to 10:90) to yield compound 6 (63.54 mg, 78%). 1H NMR (400 MHz, Methanol-d4) δ 7.78 (d, J = 7.8 Hz, 2H), 7.63 (dd, J = 7.5, 4.1 Hz, 3H), 7.37 (s, 3H), 7.29 (d, J = 7.5 Hz, 2H), 7.24 – 7.21 (m, 3H), 7.18 – 7.15 (m, 2H), 7.07 (d, J = 8.2 Hz, 2H), 7.01 (d, J = 8.2 Hz, 2H), 6.78 (s, 2H), 6.17 – 6.13 (m, 1H), 6.00 (d, J = 10.7 Hz, 1H), 4.50 (dd, J = 8.3, 4.2 Hz, 1H), 4.43 – 4.36 (m, 3H), 4.32 (d, J = 6.9 Hz, 2H), 4.21 (q, J = 7.0, 6.6 Hz, 2H), 3.89 (d, J = 2.9 Hz, 3H), 3.80 (dd, J = 11.2, 4.7 Hz, 1H), 3.50 (s, 4H), 3.39 (t, J = 5.2 Hz, 2H), 3.06 (s, 4H), 2.18 – 1.79 (m, 11H), 1.42 – 1.39 (m, 3H), 1.33 (d, J = 6.1 Hz, 3H).

Compound FITC-N201-ser-gal

A solution of compound 6 (63.54 mg, 0.05 mmol) in DMF, piperidine (200 µl) was added to selectively deprotection of Fmoc. After 1h for remove Fmoc, the solution is evaporated in vacuo. The residue dissolve DMF (1 ml), FITC (5 mg, 0.0125 mmol), TEA (5.2 mg, 0.5 mmol) and stirred overnight. The solution was evaporated in vacuo. The residue dissolve in MeOH and purified by HPLC to yield FITC-N201-ser-gal (15 mg, 24%). 1H NMR (400 MHz, Methanol-d4) δ 7.76 – 7.67 (m, 3H), 7.25 – 7.17 (m, 7H), 7.06 (dd, J = 23.4, 8.0 Hz, 8H), 6.91 (d, J = 2.3 Hz, 1H), 6.83 (d, J = 9.2 Hz, 3H), 6.77 (d, J = 6.5 Hz, 1H), 6.18 (d, J = 10.8 Hz, 2H), 6.02 (d, J = 10.8 Hz, 1H), 3.98 (t, J = 5.2 Hz, 3H), 3.94 (s, 3H), 3.81 – 3.77 (m, 3H), 3.33 (s, 1H), 3.18 (q, J = 5.8, 4.9 Hz, 4H), 1.73 (dd, J = 20.6, 5.9 Hz, 7H), 1.44 (d, J = 6.0 Hz, 3H), 1.38 (d, J = 6.0 Hz, 3H).

1. NMR spectra

Figure S1. ^1^HNMR of Compound 2 (N201)


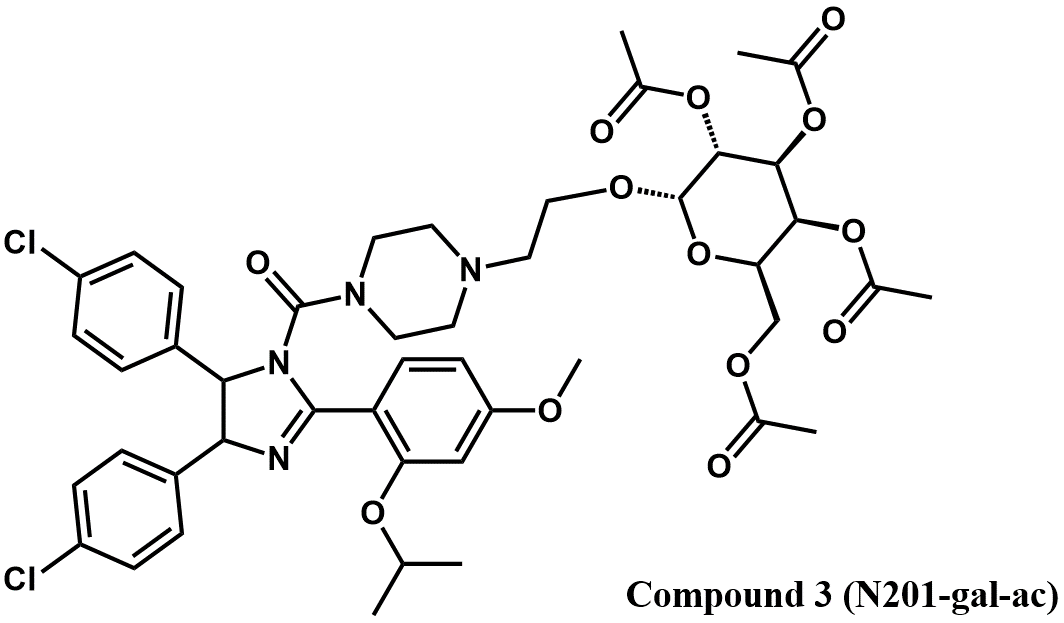


Figure S2. ^1^HNMR of Compound 3 (N201-gal-ac)


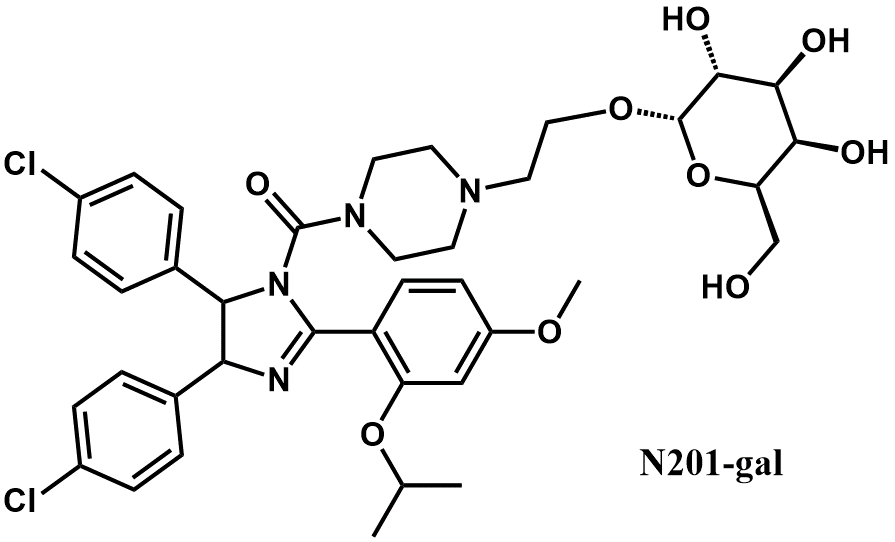


Figure S3. ^1^HNMR of Compound N201-gal


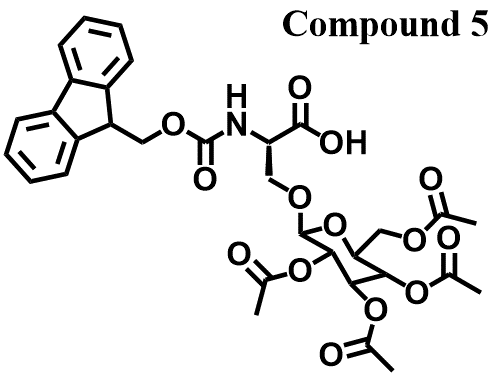


Figure S4. ^1^HNMR of Compound 5 (Fmoc-gal-ac)


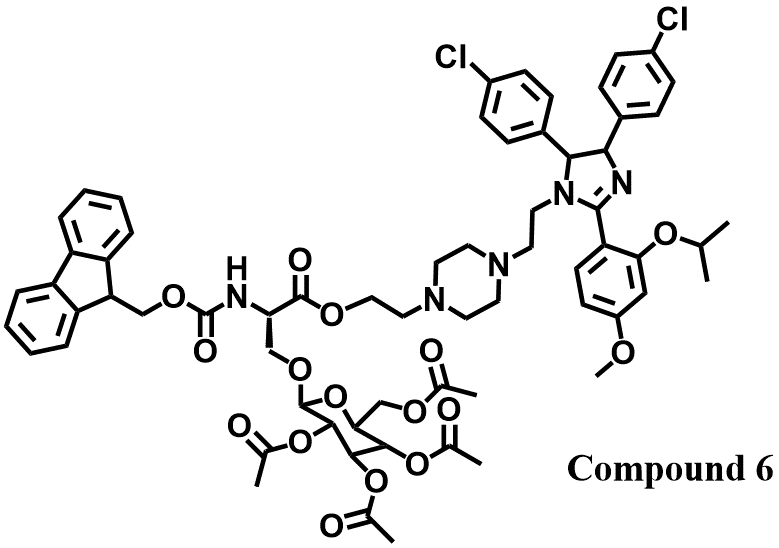


Figure S5. ^1^HNMR of Fmoc-N201-gal-ac

Figure S6. ^1^HNMR of Compound FITC-N201-gal

1. Supplementary figures


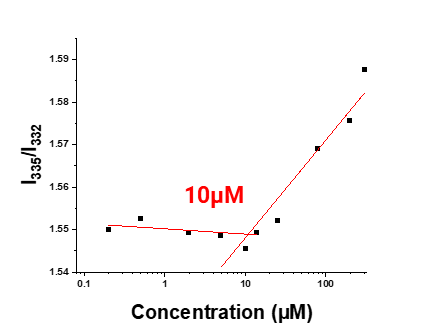


Figure S7. CMC of N201-gal by using pyrene assay

Figure S8. DLS of N201-gal 0.5wt% NaCMC for oral administration.

Figure S9. HPLC analysis of stability N201-gal in 55% serum in PBS.


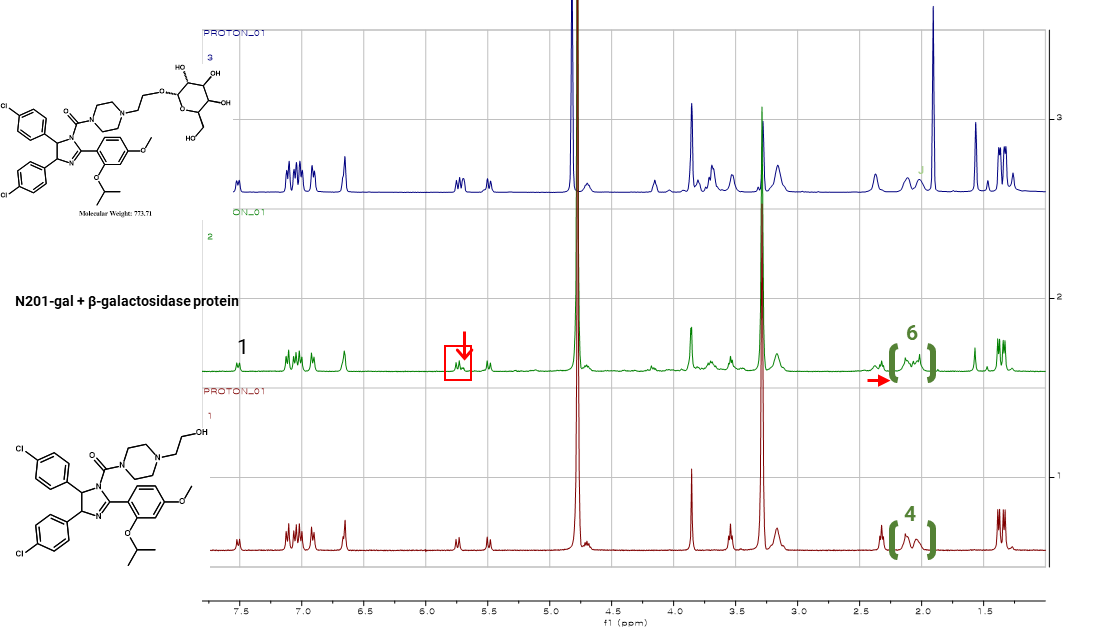


Figure S10. ^1^HNMR of β-galactosidase cleavage assay N201-gal to N201


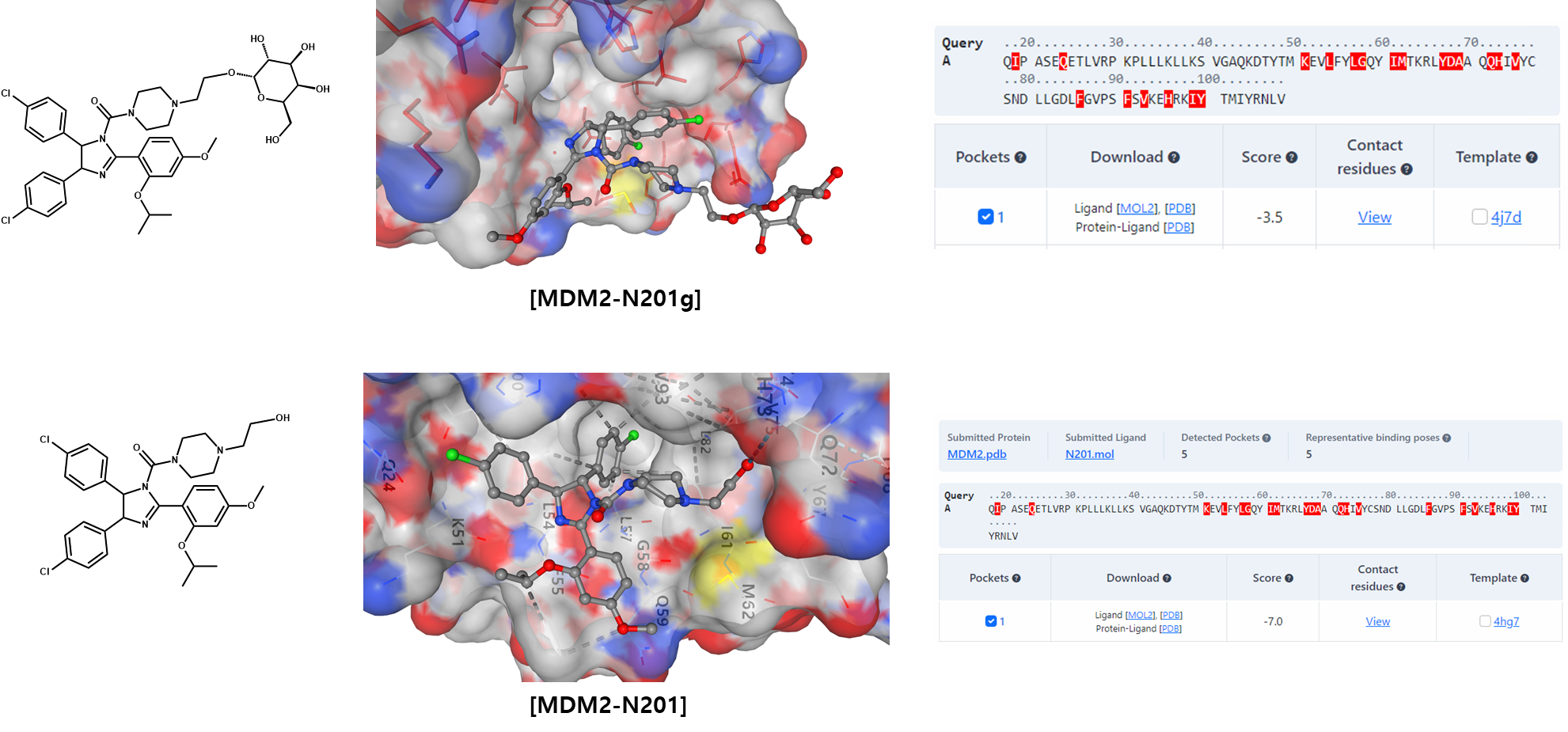


Figure S11. Docking simulation with N201-gal and N201 with MDM2 protein.


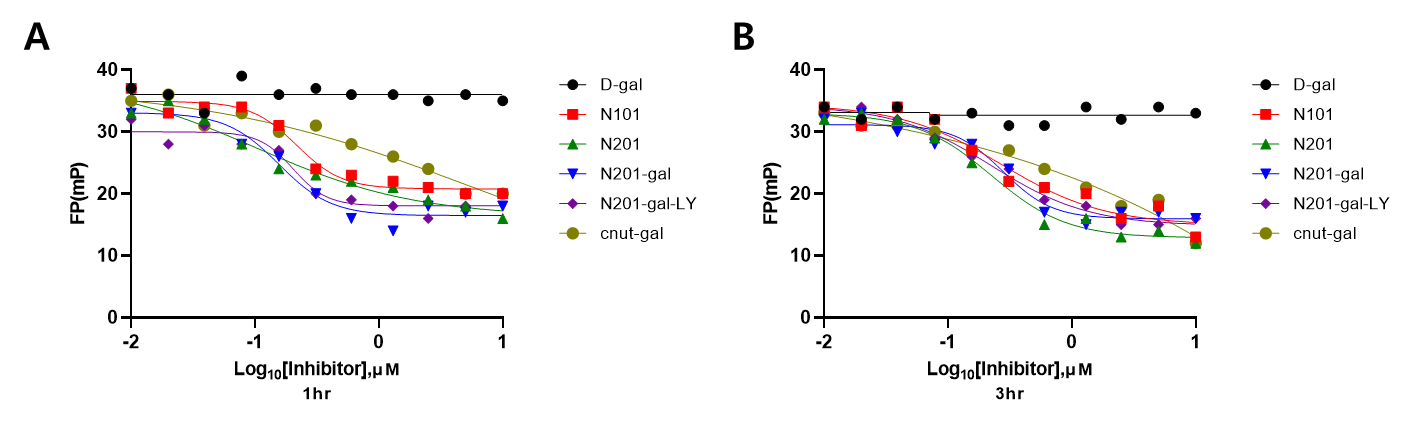


Figure S12. FP analysis of MDM2 inhibition with Nutlin-3a, N201, N201-gal.


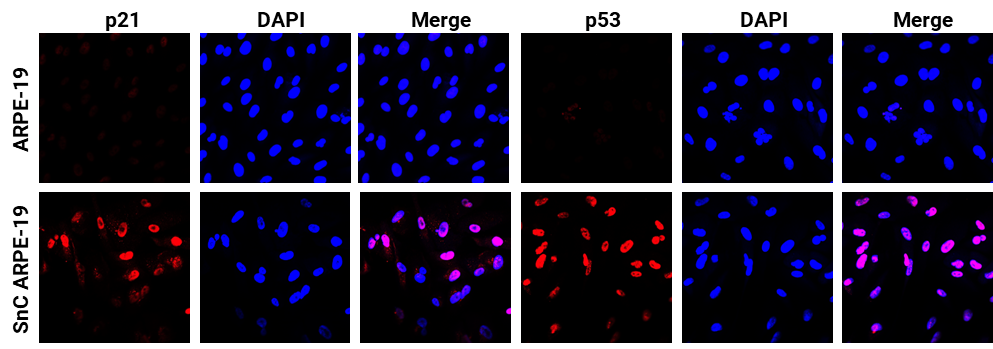


Figure S13. Immunostaining of ARPE-19 and SnC ARPE-19 with p21, p53 for senescence marker.


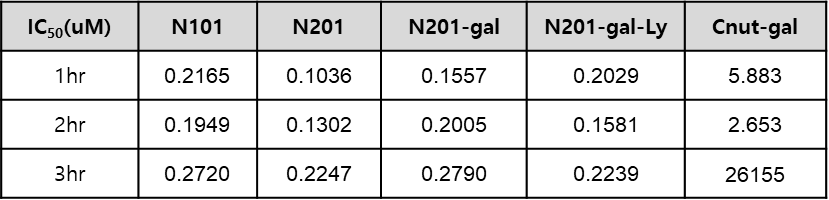


Table S1. FP IC_50_ value of Nutlin series depending on time.


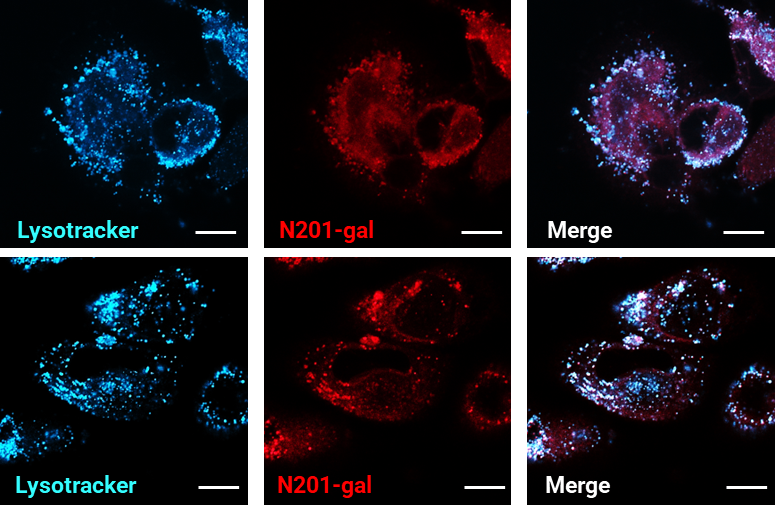


Figure S14. Organelle colocalization IR780@ N201-gal with Lysotracker in SnC ARPE-19 cell line. Scale bar = 10µm


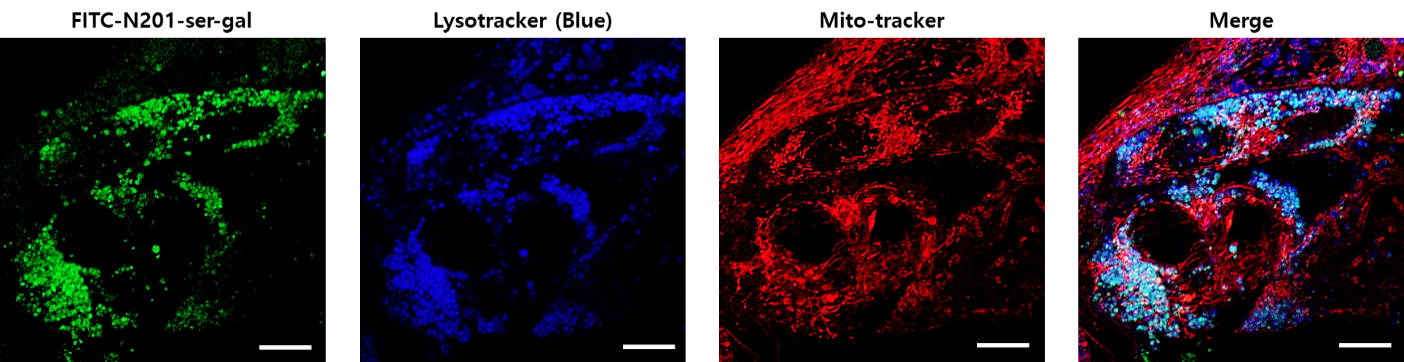


Figure S15. Organelle colocalization FITC-N201-gal with Lysotracker in SnC ARPE-19 cell line. Scale bar = 10 µm


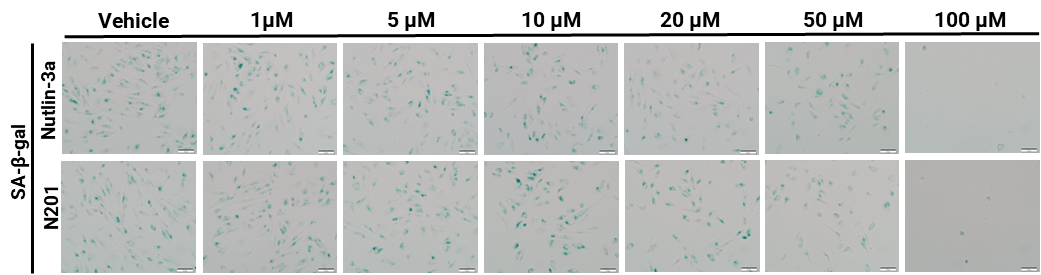


**Figure S16.** X-gal assay of **Nutlin-3a** and **N201**.

**
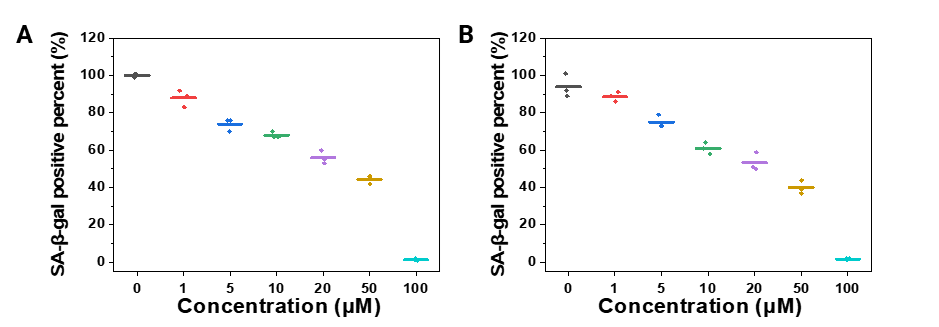
**

**Figure S17.** SA-β-gal positive percent (A) **Nutlin-3a** (B) **N201**.

**
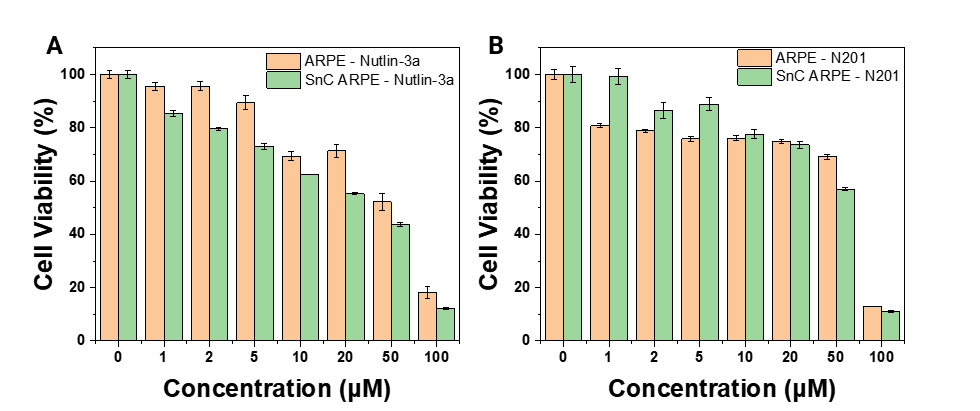
**

**Figure S18.** Cell Viability test in ARPE-19 cell and SnC ARPE-19 cell line. (A) **Nutlin-3a** (B) **N201.**


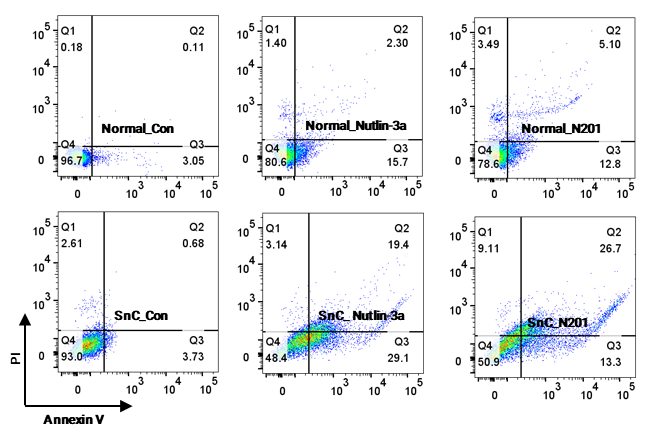


**Figure S19.** Cell Apoptosis with Annexin V-PI **Nutlin-3a** and **N201**.


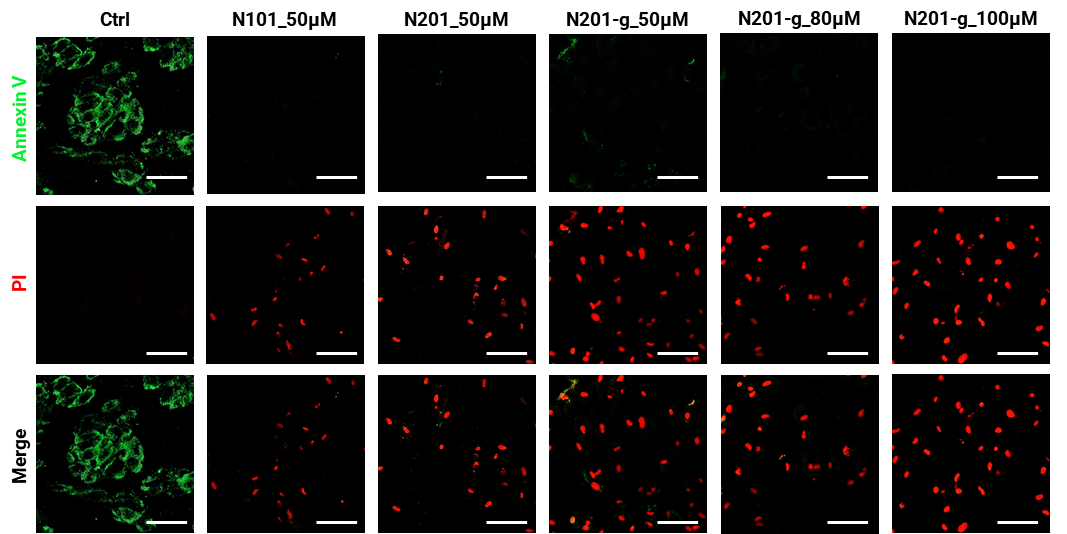


Figure S20. Calcein AM - PI assay with cell live – death for using confocal microscopy.
